# scSpecies: enhancement of network architecture alignment in comparative single-cell studies

**DOI:** 10.1186/s13059-025-03866-2

**Published:** 2025-11-20

**Authors:** Clemens Schächter, Maren Hackenberg, Martin Treppner, Hanne Raum, Joschka Bödecker, Harald Binder

**Affiliations:** 1 Institute of Medical Biometry and Statistics (IMBI), Faculty of Medicine and Medical Center, University of Freiburg, Freiburg im Breisgau, Germany; 2 Neurorobotics Lab, Department of Computer Science, University of Freiburg, Freiburg im Breisgau, Germany; 3 Intelligent Machine-Brain Interfacing Technology (IMBIT), BrainLinks-BrainTools, University of Freiburg, Freiburg im Breisgau, Germany; 4 Freiburg Center for Data Analysis, Modeling and AI, University of Freiburg, Freiburg im Breisgau, Germany; 5 CIBSS, Centre for Integrative Biological Signalling Studies, University of Freiburg, Freiburg im Breisgau, Germany

**Keywords:** Cross-species alignment, Model organisms, Deep learning, Transfer learning, Variational autoencoder, Single-cell RNA sequencing, Comparative genomics

## Abstract

Animals can provide meaningful context for human single-cell data. To transfer information between species, we propose a deep learning approach that pre-trains a conditional variational autoencoder on animal data and transfers its final encoder layers to a human network architecture. Our approach then aligns latent spaces by leveraging data-level and model-learned similarities. We utilize this for label transfer and differential gene expression analysis in cross-species pairs of liver, adipose tissue, and glioblastoma datasets. Our results are robust even when gene sets differ, or datasets are small. Thus, we reliably exploit similarities between species to provide context for human single-cell data.

**Supplementary Information:**

The online version contains supplementary material available at 10.1186/s13059-025-03866-2.

## Background

Model organisms are crucial in advancing biomedical research by offering advantages such as easy genetic manipulation and access to datasets from a variety of experimental contexts [1]. As a popular choice, mouse models have substantially contributed to the study of human diseases [2], including diabetes [3], glioblastoma [4], and non-alcoholic fatty liver disease [5]. However, translating experimental findings to humans can be challenging due to biological differences between species. Efforts to bridge this evolutionary gap include engineered mouse models that replicate human biology more closely [6]. The emergence of single-cell RNA sequencing (scRNA-seq) has also opened up opportunities for deep learning approaches to compare and contrast experimental findings across species.

Transfer learning techniques have established themselves as powerful tools for sharing information between scRNA-seq datasets. They leverage knowledge from a dataset gained during a pre-training phase to improve learning in a related dataset. These approaches often use encoder-decoder architectures to compress high-dimensional observations into a low-dimensional latent representation. Examples include Cell BLAST [7] and ItClust [8], which annotate and cluster cells based on knowledge transfer from reference datasets.

Architecture surgery techniques [9] are transfer learning techniques that aim to map single-cell data onto a pre-trained latent representation. To achieve this, they extend batch-effect covariate spaces to incorporate unseen batch effects. Specifically, additional neurons are inserted into the input layers of the encoder and decoder networks to represent batch effects for the query dataset. All other weights remain fixed during subsequent fine-tuning. Architecture surgery is used by a diverse set of models, enabling integration of varied datasets [9–12]. Despite the method’s success, two primary challenges remain unaddressed for datasets from different species.

First, some genes lack orthologs in other genomes, which requires different interpretations of certain input nodes in their neural network architectures. For example, 20% of human protein-coding genes and a substantial percentage of small and long non-coding RNAs lack one-to-one mouse orthologs [13]. To enable training, architecture surgery-based approaches restrict training to orthologous genes or zero-fill missing values. Outside of architecture surgery, models like SATURN [14] match orthologous and paralogous gene groups via protein sequences with transformer-based language models to macrogenes. However, non-homologous genes are still excluded during integration.

The second challenge is that functional or phenotypic similarities between cells do not always translate into similar gene expression patterns, which can vary significantly between species [13]. Therefore, neural networks may struggle to recognize similar cells. Both challenges combined can result in misaligned representations when transfer learning or architecture surgery techniques are applied to cross-species dataset pairs.

To account for differences in gene sets, expression profiles, and species-specific characteristics, we introduce scSpecies. The goal of cross-species integration by scSpecies is to learn a shared representation of datasets that allows for the identification of biologically similar cells, i.e., cells that execute comparable functions across different species. This facilitates various downstream analyses, including annotation matching and label transfer, identification of homologous cell types, and differential gene expression analysis.

Our approach enables this by aligning architectures across datasets from different species. By architecture alignment, we refer to the process of modifying pre-trained network architectures such that functionally similar cells across species are mapped to similar outputs. Therefore, aligned encoder architectures return a unified low-dimensional representation of datasets across species. Our approach pre-trains a conditional variational autoencoder-based model [15] and fully reinitializes the encoder input layers and the decoder network during fine-tuning. Architecture alignment in our approach is guided by a nearest-neighbor search performed on homologous genes, which estimates similarity between cells in both datasets.

This incentivizes our model to map biologically related cells into similar regions of the latent space. The neighbor search requires only a subset of observed genes to be homologs, while all remaining genes can have no relationship at all. Moreover, scSpecies enables nuanced comparisons of gene expression profiles by generating gene expression values for both species from a single latent variable.

We tested our method on data from various species and organs, including liver cells [16], white adipose tissue cells [17], and glioblastoma immune response cells [18]. Our results demonstrate that scSpecies effectively aligns network architectures and latent representations. We improve upon cell-type label transfer from the initial nearest-neighbor search and existing approaches for cross-species alignment when measured in terms of accuracy and multiple clustering metrics.

## Results

We present scSpecies, a tool for researchers who wish to use one scRNA-seq dataset as a context for another from a different species. In the following, the dataset of the model organism is referred to as the ‘context dataset’, and the dataset of the target organism is referred to as the ‘target dataset’. scSpecies learns a unified low-dimensional representation of both datasets, enabling transfer of information and the analysis of similarities and differences between the datasets.

Besides the context and target datasets, the model requires a sequence containing indices of homologous genes, indicator variables for experimental batch effects, and cell-type or cluster labels for the context dataset. Although specific cell-type annotations for the target dataset are not required, alignment should be performed with a context dataset that is comprehensive enough so that it contains suspected cell types of the target dataset.

**Fig. 1:**
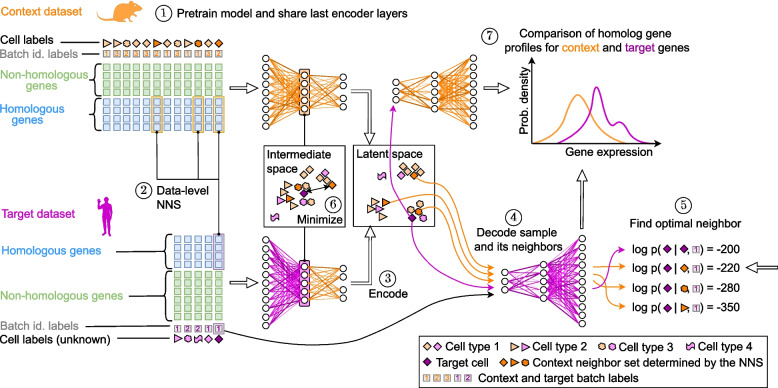
Graphical representation of the scSpecies workflow. The model is trained in a pre-training and fine-tuning phase. First, a pre-training phase trains the context encoder and decoder neural networks (Step 1). Afterwards, a *k*-nearest-neighbor search is performed on shared genes of the context and target dataset. This identifies a fixed set of *k* context neighbors for every target cell (Step 2). During fine-tuning, the weights of the last encoder layers are incorporated into the encoder model for the target species. Then, target cells are encoded into the latent space (Step 3). Without adjustment, the model might learn a misaligned representation of context and target datasets. Therefore, the model is incentivized to align target cells with a similar cell of its neighbor set. For cells with high agreement among cell labels of their neighbors, we decode each neighbor’s latent variable with the target decoder, conditioning on the human batch label, to define the corresponding target-decoder distribution (Step 4). The optimal candidate for alignment is chosen as the neighbor whose latent representation resulted in the highest log-likelihood when sampling the target cell from the respective target decoder distribution. This identifies the most similar context cell given the learned latent manifold (Step 5). The distance between the optimal candidate and the intermediate representation of its target cell is minimized (Step 6). After training, the model returns an aligned latent representation through which labels and information can be transferred. Additionally, normalized gene expression profiles can be compared by decoding latent variables with both decoder networks (Step 7)

The proposed workflow (see Fig. 1) aligns network architectures of two single-cell variational inference (scVI) [19] models using a pre-training strategy. In scVI, encoder neural networks map gene expression vectors into a compressed latent space, separating cells by biological features while removing technical artifacts from experimental batch effects or different library sizes. Conversely, a decoder maps from this low-dimensional representation onto parameters of a negative binomial distribution to (re-)generate gene expression data.

First, our proposed approach pre-trains an scVI model on the context dataset. Afterwards, its last encoder layers are transferred into a second scVI model for the target species. The aim of this architecture transfer is to share learned information within the network weights between datasets and species. During subsequent fine-tuning, the shared encoder weights remain frozen while all other weights are optimized.

Unlike existing architecture surgery approaches, we therefore align architectures in a reduced intermediate feature space instead of at the data-level, which allows us to incorporate different input features between datasets. This approach is inspired by the notion of mid-level features from the field of computer vision [20, 21]. These represent abstractions of the input image learned by neural networks in their intermediate layers. Mid-level features combine individual elements of the input space into more general structures, such as contours, specific shapes, or parts of objects. Transfer learning approaches then retrain the last layers to transition these intermediate representations into task-specific network outputs for different datasets [22].

Unlike images, scRNA-seq datasets lack ordered patterns, as gene expression vectors can be permuted without changing their information content. Nevertheless, the first encoder layers translate dataset-specific features, such as influences of experimental batches or interactions between observed genes, into a higher abstraction level. The resulting intermediate representation integrates these data-specific features and is less susceptible to noise and systematic differences between species, such as different gene sets. (For more details about the intermediate feature spaces of scVI and scSpecies models, see Additional file 1: Fig. S1.)

To link the reinitialized encoder layers with the pre-trained structure, we guide alignment through a data-level nearest-neighbor search. Specifically, we identify sets of similar cells using cosine distance on log1p-transformed counts of homologous genes. Afterwards, scSpecies minimizes the distance between an intermediate target cell’s representation and a suitable candidate from its set of nearest neighbors. The model determines the most suitable context cell dynamically during fine-tuning as the candidate whose latent representation, decoded by the target decoder, yields the highest log-density value for the target cell’s gene expression values within the target decoder’s distribution. To counter misclassifications that may have occurred on the data-level, we align mid-level features only for target cells whose context neighbors have high agreement among their cell labels.

During model fitting, we thus utilize similarity information both at the original data-level and at the level of learned features. The aligned latent space then captures cross-species similarity relationships based on the fitted model, which facilitates information transfer across species.

### scSpecies returns an aligned cross-species representation that can be used for annotation transfer

We applied the scSpecies workflow to three mouse-human dataset pairs containing liver cells, white adipose tissue cells, and immune response cells to glioblastoma. First, we observed that the alignment procedure impacted the reconstruction quality of the target decoder network only slightly (See Additional file 1: Fig. S2 and Fig. S3). On the liver cell atlas, a vanilla scVI model achieved average log-likelihood values of -1151.7 for the human dataset, while an aligned scSpecies target decoder achieved a slightly worse value of -1158.9 (higher is better).

**Fig. 2:**
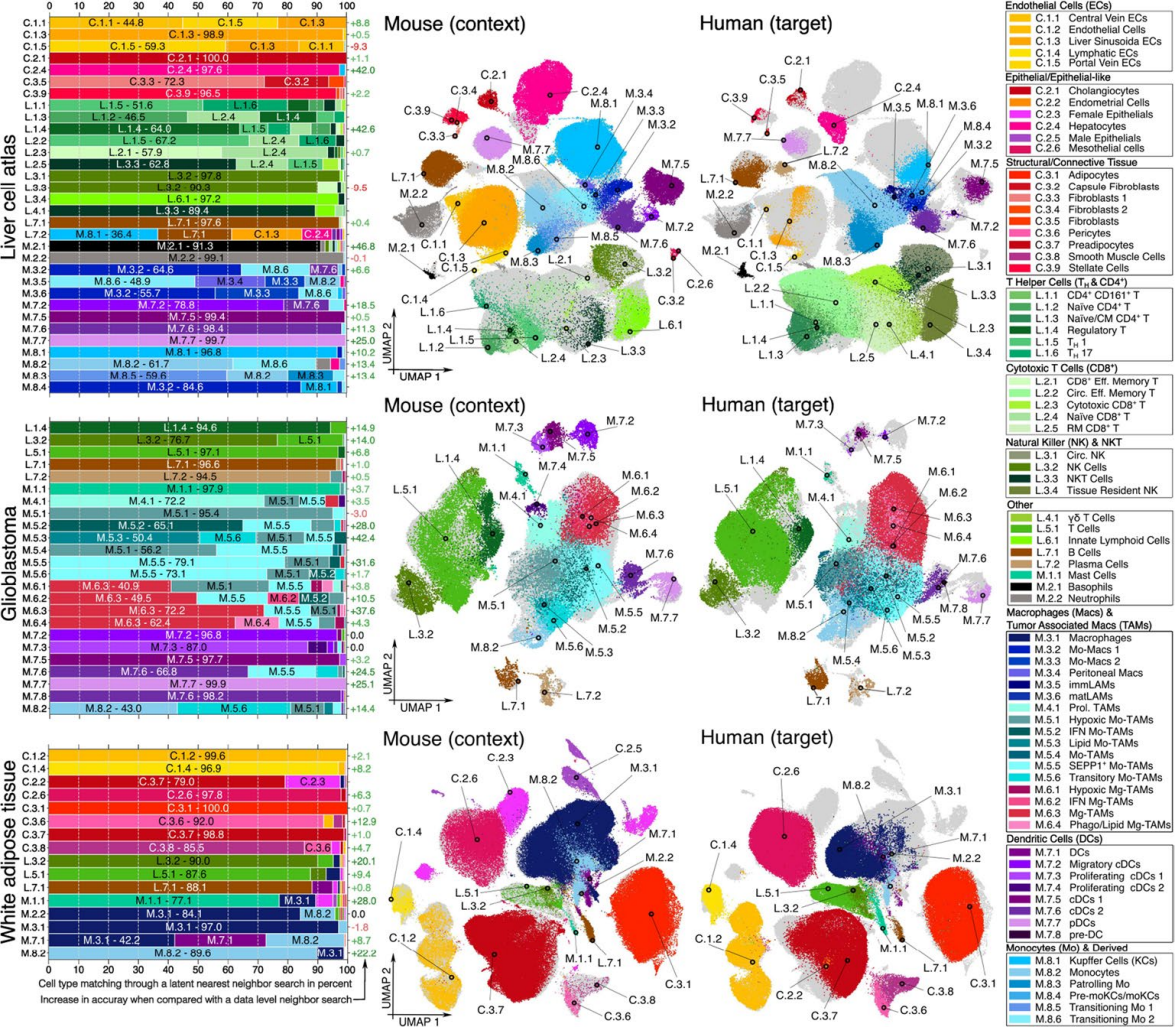
Visualization of aligned representations for three dataset pairs (liver, glioblastoma, and adipose tissue) obtained by training scSpecies with a set of 25 neighbors. On the left, bar plots indicate the accuracy of cell-type label transfer through a latent nearest-neighbor search. The left y-axis labels indicate cell-type codes corresponding to human cell labels. These codes are referenced in the legend. The bars contain the frequency of assigned mouse cell labels through a neighbor search in the latent space. The right y-axis labels indicate improvement in accuracy for shared cell types when compared with a data-level nearest-neighbor search. The center panel visualizes UMAP embeddings of the aligned latent representations. The cells from the other dataset are indicated in light gray

Next, we visually examined alignment through UMAP embeddings [23] of the combined latent representation (Fig. 2). The UMAP plots indicate alignment of similar cell types across species in the latent space. Cell types without context counterparts aligned with related cell types or formed distinct clusters.

To further investigate alignment quality, we inferred target cell labels using a latent nearest-neighbor search. Correct label transfer from context to target cells through their local latent neighborhood region indicates a well-structured latent space. scSpecies internally defines a similarity measure for two target and context cells by subtracting the log-likelihoods that are obtained by evaluating the target decoder distribution of both latent representations at the target cell’s gene expression vector. We use this measure to perform a nearest-neighbor search in the latent space and transfer a cell-type label from context to target dataset via majority voting among the 25 nearest neighbors. Figure 2 contains cell-type-wise percentages of inferred context cell labels. We observed accurate label transfer in most cases, while classification errors occurred mostly within similar cell types.

Label annotations in the datasets have a broad label category and a fine label category containing cell subtypes. Averaged over ten random seeds, we obtained label transfer accuracies balanced across cell types of different sizes of 92% and 73% for the liver, 89% and 67% for the glioblastoma, as well as 80% and 49% for the adipose tissue dataset for broad and fine cell-type labels, respectively.

These values represent considerable improvements over label transfer using the data-level nearest-neighbor search and CellTypist [24] for cell-type annotation. Next to the bar plots, Fig. 2 contains the cell-type-specific differences in accuracy of annotating labels using scSpecies compared with the data-level neighbor search. For fine cell-type annotations, accuracy increased by 11% absolute, on the liver cell atlas, by 10% on glioblastoma data, and by 8% for the adipose tissue dataset. CellTypist struggled to transfer labels on cross-species datasets and achieved accuracy scores of 38% (-35%), 42% (-25%), and 41% (-8%) on the three dataset pairs for fine label annotation.

We observed a greater increase in label transfer accuracy for cell types with noisy data-level nearest-neighbor sets but a clear separation in their pre-trained latent space. For example, the initial neighbor search matched less than half of all human liver basophils (cluster M.2.1) with mouse counterparts. This value improved to over 90% through our method. However, for cell types where both the context scVI model and the neighbor search failed to differentiate clusters (as seen for dendritic cells, monocytes, and macrophages in the adipose tissue dataset), scSpecies was also unable to achieve proper separation. Furthermore, for cell types with noisy neighbor search results, specifically liver hepatocytes and portal vein endothelial cells, misclassifications of the entire cell type occurred in one random seed.

### scSpecies’ similarity measure guides alignment and can be used to match cell types

To investigate how scSpecies aligns target cells with their preferred match during training, we created context and target prototype cells consisting of empirical median gene expression values within a cell type.

**Fig. 3:**
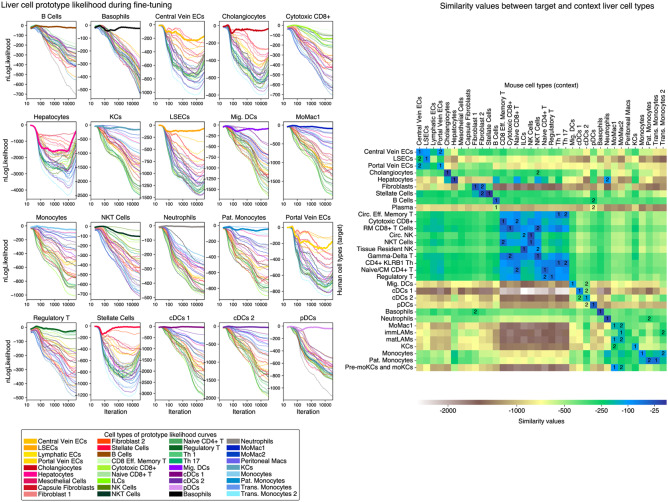
(Left) Illustration of the alignment process of scSpecies with $k=25$ neighbors on the human liver cell atlas. On the y-axis, we plot differences in log-density values derived from reconstructing human liver cell prototypes both from the set of mouse prototype latent variables and the target prototype latent variable. The reconstruction graph of the respective cell type is highlighted boldly. The x-axis shows these values at a training iteration, averaged over the last 5% of iterations and ten random seeds. (Right) Heatmap showing averaged similarity values between mouse context and human target cell types in the liver cell dataset. Similarity scores within each target cell type to each context cell type are represented row-wise by colors, and the two most similar context cell types for each target cell type are indicated by numbers

During alignment, we tracked the log-likelihood differences resulting from decoding target prototypes from the context prototypes’ latent representation and its own latent representation (Fig. 3). At the outset, the likelihoods for all prototypes were nearly equal. This resulted in alignment driven by chance favoring alignment with context candidates of the most occurring cell label. For cell types with a noisy neighbor set, corrections during later training stages eventually aligned them with appropriate counterparts by assigning higher log-likelihood values. We observed this with hepatocytes, migratory DCs, and basophils, which had nearest-neighbor search accuracies of 56%, 61%, and 45%, respectively.

After alignment, we used the similarity measure defined by scSpecies to assess the similarity between target and context cell types. (See the “Measuring the similarity of context and target cells” section in Methods for details). We observed that most homologous cell types across species are correctly assigned a high similarity score (see Fig. 3 and Additional file 1: Fig. S4). Therefore, the internal similarity measure can be used to match annotation labels across species and potentially uncover homologous cell types between model organisms and target species. We observed mismatching by scSpecies for some sub-cell types, all of which were severely underrepresented in the dataset, and their neighbor search yielded predominantly incorrect results. For example, in the liver cell dataset, cytotoxic CD8$^+$ cells consisted of 0.1% of the context dataset size and were matched with 1% at the data-level, while natural killer T cells made up 0.8% of the context dataset size and had an initial data-level NNS accuracy of only 11%. Both of these cell types were matched with other T-cell subpopulations.

### scSpecies compares favorably with other methods and performs well on small datasets

To benchmark our method, we compared scSpecies against several CVAE-based alignment approaches. We included a vanilla scVI [19] model trained on the combined context and target datasets. Building on an scVI base model, we included scArches [9] and scPoli [10], architecture surgery-based approaches that map the human target dataset into a pre-trained context latent space by extending its experimental batch effect covariate spaces. scPoli learns new batch covariate representations from data and pulls cells towards prototype cells in the aligned latent space. Additionally, we included sysVI [25] which replaces the Gaussian prior with a VampPrior and adds a cycle-consistency loss to enforce consistency between the context and target latent spaces. These models require matching gene sets and are therefore only trained on the set of shared homologous genes. Finally, we evaluated SATURN [14], which leverages transformer-based gene matching to align homologous and functionally equivalent genes across species. After pre-training, target cell annotations can be utilized to guide the alignment process. We trained two varitants, one that utilizes matched context and target cell-type labels during fine-tuning and one without matching annotations, which is equivalent to label information that our approach requires. Here we provided SATURN with Leiden cluster information. For scSpecies, we experimented with different nearest-neighbor set sizes ($k=1$, $k=25$, and $k=250$) and alignment in the intermediate feature and latent space.

Alignment quality was assessed using a set of metrics established by [26, 27]. Four metrics capture species mixing in the latent space, and four metrics evaluate the preservation of biological structure in the aligned representation. The respective metrics are aggregated into a species mixing score and biology conservation score, which in turn define an integrated score by weighting species mixing by 0.4 and biological conservation by 0.6.

**Fig. 4:**
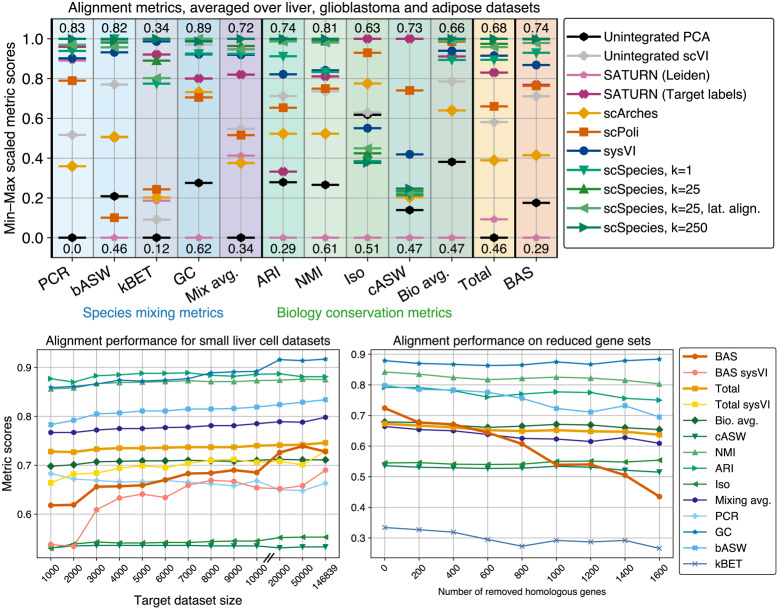
Comparison with other dataset alignment approaches (top) and performance of scSpecies on small datasets (bottom left) and reduced shared features (bottom right). Higher scores indicate better performance. Exact unscaled values and standard deviations are provided in Additional file 2: Table S1-S3. Results are averaged over ten random seeds. (Top) Alignment performance averaged across liver, adipose, and glioblastoma datasets for different alignment approaches across species mixing metrics (blue background), biology conservation metrics (green background) and balanced label transfer accuracy (ochre background). Metrics are min-max scaled but their unscaled max and min values are provided above and below the best and worst performing model. (Bottom left) Alignment performance for small liver cell target datasets. Metrics are not min-max scaled to display their absolute changes. The target dataset was randomly sampled to contain between 1,000 and 50,000 human liver cells. We additionally plot the label transfer performance and total alignment score performance for sysVI. (Bottom right) Alignment performance for a reduced homologous gene set. Metrics are not min-max scaled. The shared gene sequence of length 1,808 was gradually reduced by 200 to 1,600 genes

Our experiments (Fig. 4, top panel), reveal that scSpecies achieved superior species mixing performance compared to the alternative approaches, indicating a more harmonized latent space across different species. In terms of biological conservation, scSpecies outperformed other methods on two of the four metrics. Specifically, scSpecies achieved high performance on the Adjusted Rand Index and Normalized Mutual Information comparing latent Leiden clusters and cell-type labels, though scSpecies scored lower on isolation scores, which measure separation of latent cell-type clusters that are unique to one of the two species. We attribute this to the observation that unique target cells tend to align with related context cells, rather than forming distinct clusters. Overall, the integrated score of scSpecies with $k=25$ neighbors was 0.678, which exceeds the scores of sysVI (0.665), SATURN with matching annotation (0.647), scPoli (0.609), scVI (0.591), and scArches (0.548).

For cell-type label transfer, scSpecies with $k=25$ neighbors achieved the highest balanced accuracy of 73.5%, followed by sysVI (67.6%), a data-level neighbor search (64.7%), SATURN with matching annotation (63.2%), scPoli (62.9%), scVI (60.6%), CellTypist (50.6%), and scArches (47.5%). UMAP representations of aligned latent spaces can be found in Additional file 1: Fig. S5 and Fig. S6

Analyzing the impact of different neighbor search set sizes, we observed that using a small neighbor set containing only a single neighbor forced the model to align some target cells with suboptimal matches, as the approach could not correct for noisy results obtained on the data space. This resulted in lower performance across almost all metrics. Increasing the neighbor set to $k=25$ improved alignment across datasets, whereas further enlarging the set to large sizes of $k=250$ neighbors yielded only minimal additional gains. However, we observed a reduced alignment performance of 6% for cell types smaller than the amount of neighbors, which are underrepresented in neighbor sets of homologous target cells. Furthermore, we examined an alternative strategy of directly aligning the latent representations rather than aligning intermediate features. This approach resulted in a slight reduction in label transfer accuracy and the integrated score of around 1-2%. Still, we see direct latent alignment as a viable alternative for scenarios where only the resulting context latent representation can be shared and the context model weights or the context gene expression data are inaccessible.

We also tested scSpecies performance in a scenario where the target dataset was small but equally diverse in terms of cell types and batch effects (Fig. 4, bottom left panel). Specifically, we sampled 1,000–50,000 cells from the human liver dataset and aligned this reduced target dataset with the full mouse context dataset in 15 repetitions. We observed a gradual drop in performance score for smaller datasets. When measured in label transfer accuracy (red line), a noticeable drop in accuracy occurred between 20,000 and 10,000 cells. Despite a performance drop when compared to larger datasets, the model was still able to reasonably align smaller target datasets. We attribute this to the nearest-neighbor component of scSpecies. As the nearest-neighbor search achieves its matching regardless of the target dataset size, it can guide alignment in cases where target data is scarce.

Finally, we tested the amount of shared features between the datasets that are required for meaningful alignment by removing 200–1,600 genes from the 1,808 shared genes from the target dataset. Again, we observed a gradual reduction in label transfer accuracy and species mixing and biology conservation scores, while observing a steep drop in label transfer accuracy for datasets with fewer than 1000 shared genes (Fig. 4, bottom right panel). The reduction in performance can be attributed to progressively noisier nearest-neighbor searches whose accuracy decreased from 62% for the original matching to 48% with a gene set size of 1000 shared genes to 30% for only 200 shared genes.

However, in a scenario where the shared gene set was completely removed from both the context and target datasets, but the original neighbor matching was kept, scSpecies could still achieve accurate alignment. Here, we only measured a slight decrease in balanced label transfer accuracy by 4% and a lowered integrated score of -0.03 when compared to performance on the full dataset. This suggests that the initial nearest-neighbor search matching is an important part of our approach and that the non-shared gene set is leveraged by the model, as an alignment restricted to it is still feasible.

### scSpecies can align datasets of multiple species

**Fig. 5:**
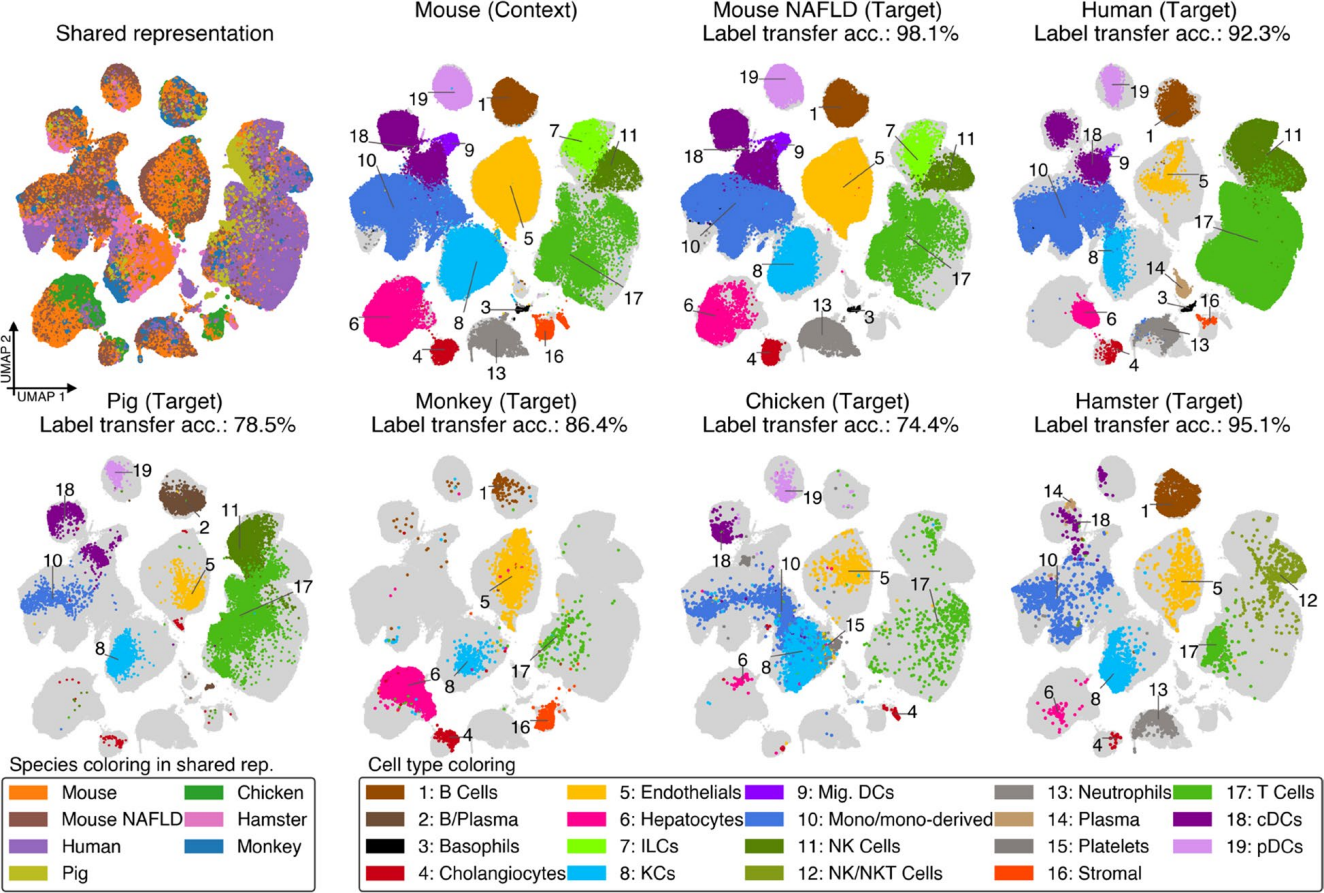
We utilized scSpecies to obtain an aligned liver cell landscape that spans multiple species. The mouse dataset serves as a context for each species. The figure shows the UMAP representation of the shared latent space. Coloring and label transfer accuracy are according to coarse cell-type labels

We employed scSpecies to simultaneously align liver cells from mice with fatty liver disease, humans, pigs, monkeys, chickens, and hamsters, using the same context model trained on healthy mice data for each target dataset (Fig. 5). We successfully obtained aligned latent representations across species, despite fewer than half of the genes having mouse orthologs in some datasets.

An intriguing application of scSpecies is the potential to align datasets with very limited gene coverage, or even when there is no overlap in the observed gene set. This can be achieved by aligning each dataset to a comprehensive context dataset that shares a common gene set with both species.

However, a limitation of this approach is its inability to align cell types not present in the context dataset. For example, plasma cells, which were absent from the mouse dataset, were not aligned across the human, pig, and hamster datasets.

### scSpecies offers insights into the genetic manifestations of cells across species

To better analyze the similarities and differences between the context and target datasets, we extended our analysis from the latent space to the data-level. Here, we compared the reconstructed gene expression profiles and assigned relevance scores to the input genes.

We decoded latent representations using both context and target decoder models to obtain normalized gene expression vectors for each species. These vectors allow us to compare and analyze the gene expression profiles of cells that have similar underlying biological properties while correcting for different library sizes that may be present between species. This analysis benefits from the correspondence between latent representations of both species, which is difficult to establish at the data-level.

**Fig. 6:**
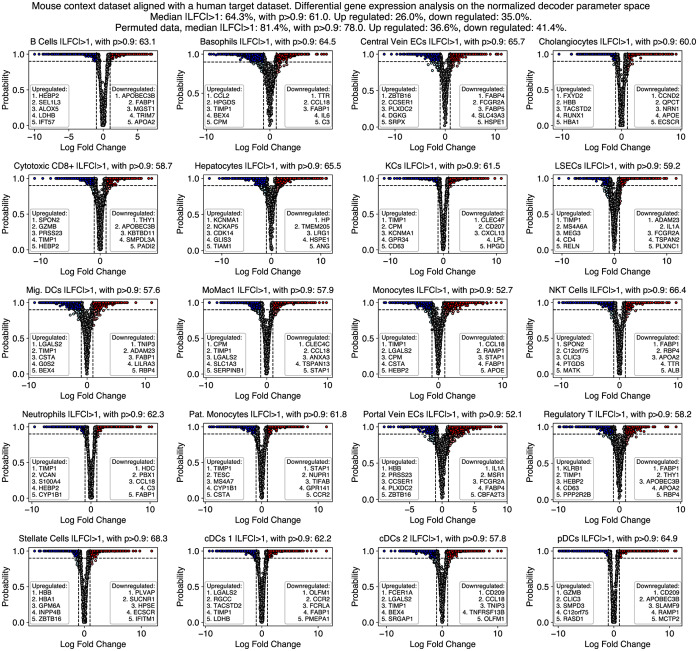
A comparative analysis of gene expression profiles between humans and mice using scSpecies. We computed the median of the empirical log2 fold change (LFC) distribution, displayed along the x-axis. The y-axis illustrates the probability of a gene being differentially expressed with an absolute LFC exceeding one within a cell type. The compared cells are decoded from a randomly selected latent value within a latent target cell-type distribution. The figure lists the top five genes in humans that are upregulated and downregulated in comparison to their mouse homologs in each shared cell type. The figure also lists the cell-type-wise fraction of genes that are differentially expressed in at least 90% of decoded samples

For our investigation, we ranked homologous genes by log2 fold changes (LFCs) on the decoders’ normalized gene expression output space and focused on cell types present in both the mouse and human liver datasets. We also calculated the probability of observing genes as differentially expressed when sampling from the latent distribution of a cell type (Fig. 6). First, we found LFC values highly correlated when compared with a data-level analysis (see Additional file 1: Fig. S7).

Averaging across cell types revealed that 64% of the genes exhibited an LFC value above one and 61% of genes were differentially expressed in more than 90% of samples. Among these, 26% of human genes were upregulated and 35% were downregulated compared with their mouse counterparts in over 90% of decoded cells. Interestingly, cell types were the data-level nearest-neighbor search struggled to find an accurate match with target cells, such as hepatocytes or plasmacytoid dendritic cells, were among those cell types with the highest ratio of differentially expressed genes. Calculating LFC values on randomly permuted genes yielded higher, although not substantially higher, LFC values, with 81% (+17%) of genes exhibiting an LFC greater than one.

For white adipose tissue datasets, 71%, and for glioblastoma datasets, 54% of genes exhibited an LFC value greater than one. We compared mouse-human LFC ratios with values observed at the context-target dataset pair of healthy mice and mice with liver disease as shown in Additional file 1: Fig. S8. Here, only 20% of genes had an LFC value above one. Calculating DGE values on a random gene permutation yielded a fraction of 80% (+60% absolute) of differentially expressed genes. Substantially lower LFC values for cross-species datasets highlight the differences in gene expression patterns across species.

We extended our study by calculating relevance scores via layer-wise relevance propagation (LRP) [28]. These scores measure each gene’s contribution to a cell’s latent value, offering insights into the learned significance of specific genes across different cell types and species. LRP was recently used to explain neural network predictions on scRNA-seq data [29] and results are shown in Additional file 1: Fig. S9.

First, we found no substantial difference in relevance scores between non-shared and shared genes in the datasets, suggesting that training only on the shared gene set withholds an informative part of the data that is used by the target model to derive its latent representation. Second, we found that the relevance scores were correlated with the gene expression levels. For the mice and human liver datasets, we found a Spearman’s $\rho$ between the expression level of genes and their relevance scores of 0.67 and 0.69 and a Pearson correlation coefficient of 0.63 and 0.71. This suggests that highly expressed genes become relevant features for the neural networks. A gene with high relevance scores across most cell types was *MALAT1*, which is highly conserved across mammals [30].

## Discussion

We introduced scSpecies, a novel deep learning approach designed to align neural network architectures across different species. Aligning architectures on cross-species datasets has been a challenging task due to technical artifacts introduced by experimental batch effects, differences in genomes between species, and variations in gene expression patterns among homologous genes. scSpecies integrates these data-level differences by aligning dataset representations in intermediate neural network layers. These intermediate features capture higher-level, biologically relevant patterns such as cell types while reducing the influence of species-specific differences, noise, and technical variations. Additionally, the incorporation of a nearest-neighbor search leverages data-based similarity information to guide the alignment process, ensuring that biologically similar cells are mapped closely in the latent space. Our results demonstrate that scSpecies effectively aligns scRNA-seq data from a diverse set of species and tissues. scSpecies compares favorably with other approaches for single-cell dataset integration and shows robust performance even when the target dataset consists only of a few thousand cells.

A limitation of scSpecies is that cell types unique to the target dataset tend to align with biologically close cell types of the context dataset instead of being identified as new clusters by the model. This could lead to misinterpretation of dataset-specific cell populations. Additionally, when creating a collection of multiple species, cell types not present in the context dataset will not align across species that do exhibit them. These limitations can be addressed by artificially enriching the context dataset with data from a cell atlas and evaluating target data that aligns with atlas-specific cell types with caution.

Another limitation is that alignment can be unreliable for rare cell types, which can require larger sample sizes for the *k*-nearest-neighbor guidance to yield high-confidence matches. When *k* exceeds the within cell-type sample size, this can cause insufficient representation of the cell type in neighbor sets used during alignment.

A further limitation is that performance of scSpecies is tied to its scVI base model. When its latent clustering performance is influenced, for example, by excessive noise in the context data, high sparsity, or distribution skewness [31], scSpecies inherits these properties, which might negatively affect analysis on the target dataset. A researcher applying scSpecies should therefore check the quality of latent cluster separation after the pre-training step.

Finally, results from data-level analyses should be treated as provisional and should be reproduced with independent experiments. Data-level analyses results from scSpecies can be distorted through technical artifacts, for example by batch effects reintroduced by the decoder. scSpecies mitigates their effect by averaging results over all experimental batches present in the datasets. However, results still might be influenced by the effects of specific batches, especially in datasets that only contain a few experimental conditions. Also, functional similarity between cell states across species cannot be determined from transcriptomic data alone, as it depends on additional molecular and physiological layers, including regulatory, proteomic, and metabolic processes. Therefore, scSpecies can provide supportive, but not conclusive, evidence of functional similarity and should be regarded as a tool to generate hypotheses, which require validation through complementary experiments.

There remain multiple potential directions for further development of our approach. While we initially tested scSpecies with an scVI base model, the approach could be adapted to other CVAE-based models in the future. Furthermore, scSpecies could be extended to handle multimodal datasets, such as those integrating scRNA-seq with protein expression data (CITE-seq). Another area of interest could be to increase latent-space stability and enhance cross-species cell-type matching in cases of extreme sparsity and skewed count distributions. For example, this could be achieved by incorporating scLENS’s random matrix theory-driven noise subtraction [31] to automatically filter out perturbation-stable embedding components before alignment. Lastly, scSpecies would also benefit from a direct metric that identifies cell types unique to the target dataset and detects cells that may be misclassified due to noisy nearest-neighbor search results.

## Conclusions

We have introduced scSpecies, a novel deep learning approach that aligns network architecture and latent representations of scRNA-seq datasets across species. By retraining the first encoder layers, our method overcomes challenges posed by non-orthologous genes and divergent gene expression patterns, enabling more accurate cross-species comparisons. By aligning datasets from multiple species, scSpecies provides a framework to better understand and compare the cellular and molecular similarities and differences of scRNA-seq datasets across species. Therefore, we envision that our method could lead to more effective translation of experimental findings from model organisms to humans.

## Methods

We summarize all recurring notation of the Methods section in Table 1. In the following, we briefly describe the scVI model, which we subsequently use as a core of our proposed approach.

**Table 1:** Summary of recurring notation used in the Methods section

Category	Symbol	Description
**Data dimensions**	*M*	Number of cells
	*N*, *H*	Number of genes, number of homologous genes
	*S*	Number of experimental batches
**Subscripts**	*C*	Context species subscript
	*T*	Target species subscript
**Indices**	*i*	Index for context cells
	*j*	Index for target cells
	$i^*$	Index for the optimal neighbor for alignment
	*g*	Index for a gene
**Random variables**	$\textsf{Z}$	Latent variable for biological variability
	$\textsf{L}$	Latent variable for technical variability in library size
	$\textsf{X}$	Random variable for data points
	$\textsf{S}$	Random variable for experimental batches
**Distributions**	$\textsf{P}_{\textsf{Z}}$	Distribution of a random variable
	$\textsf{p}_{\textsf{Z}}$	Probability density function
	$\textsf{P}_{\textsf{X}|\varvec{z}}$	Conditional distribution
**Hyperparameters**	*k*	Data-level neighbor search size
	$\beta$	KL-Divergence loss weight
	$\eta$	Alignment-term loss weight
	*p*	Top $p\%$ of cells with high label agreement among their neighbors
**Features**	$\varvec{x}$	Gene expression vector
	$\varvec{h}$	Gene expression vector restricted to homologous genes
	$\varvec{t}$	Representation in the intermediate feature space
	$\varvec{z}$	Latent representation of a gene expression vector
	$\varvec{\rho }$	Reconstructed normalized gene expression vectors
	$\varvec{s}$	Experimental batch indicator variable
	*c*	Cell-type label
**Sets**	$\mathbb {D}$	scRNA-seq dataset
	$\mathbb {S}$	Batch indicator variables
	$\mathbb {L}_k$	Latent representation of the context neighbor cells
	$\mathbb {I}_k$	Index set of *k* nearest neighbors used for alignment
	$\mathbb {J}(p)$	Index set of target cells with top $p\%$ of neighbor agreement
**Neural Networks**	$f_{\text {enc}\,\textsf{Z}}$	Cell encoder
	$f_{\text {enc}\,\textsf{L}}$	Library encoder
	$f_{\text {outer}}$	Outer cell encoder layers
	$f_{\text {inner}}$	Inner cell encoder layers
	$f_{\text {dec}}$	Decoder

### Single cell variational inference

Consider a dataset $\mathbb {D} = \left\{ \left( \varvec{x}^{(i)}, \varvec{s}^{(i)}\right) \right\} _{i=1}^{M}$ obtained through a single-cell RNA sequencing experiment. The mathematical model behind scVI [19] assumes that gene expression count vectors $\varvec{x}$, and batch indicator variables $\varvec{s}$, correspond to observations of random variables $\textsf{X}$ and $\textsf{S}$. The gene expression data distribution $\textsf{P}_{\textsf{X}|\varvec{s}}$ is conditioned on its batch effect $\textsf{S}=\varvec{s}$. This accounts for technical artifacts during data collection. Within an experimental batch, gene expression vectors are independent and identically distributed samples from $\textsf{P}_{\textsf{X}|\varvec{s}}$.

scVI models the data distribution within a parametric family. Building on conditional variational autoencoders [15], a latent variable model is introduced. The random variable $\textsf{Z}$, corresponding to the representation of a cell in the latent space $\mathbb {R}^d$, is employed to capture biological variability among cells in the dataset. The one-dimensional random variable $\textsf{L}$ with latent space $\mathbb {R}_{>0}$ accounts for technical variability due to different library sizes. Within the model, data is generated by drawing samples for $\textsf{Z}$ and $\textsf{L}$ from a prior distribution $\textsf{P}_{\textsf{Z,L}|\varvec{s}}$. Then, gene expression data is generated by drawing from the sampling distribution $\textsf{P}_{\textsf{X}|\varvec{z},l,\varvec{s}}$.

The data p.d.f. $\textsf{p}_{\textsf{X}|\varvec{s}}$ can be expressed by integrating the joint probability across the latent spaces and then applying the general product rule of probability,

1 $$\begin{aligned} \textsf{p}_{\textsf{X}|\varvec{s}}(\varvec{x})=\int _{\varvec{z}}\int _{l} \textsf{p}_{\textsf{X}|\varvec{z},l,\varvec{s}}(\varvec{x}) \textsf{p}_{\textsf{Z,L}|\varvec{s}}(\varvec{z},l)\,\textrm{d}\varvec{z}\textrm{d}l. \end{aligned}$$

To approximate this integral, scVI performs variational inference on the intractable posterior distribution $\textsf{P}_{\textsf{Z},\textsf{L}|\varvec{x},\varvec{s}}$. Therefore, the posterior probability is approximated by a variational distribution, denoted as $\textsf{Q}_{\textsf{Z},\textsf{L}|\varvec{x},\varvec{s}} \approx \textsf{P}_{\textsf{Z},\textsf{L}|\varvec{x},\varvec{s}}$. Furthermore, scVI applies a mean field approximation, where p.d.fs of both variational and prior distribution are factorized,

2 $$\begin{aligned} \textsf{q}_{\textsf{Z,L}|\varvec{x,s}}(\varvec{z},l)=\textsf{q}_{\textsf{Z}|\varvec{x,s}}(\varvec{z})\textsf{q}_{\textsf{L}|\varvec{x,s}}(l),\ \textsf{p}_{\textsf{Z,L}|\varvec{s}}(\varvec{z},l)=\textsf{p}_{\textsf{Z}}(\varvec{z})\textsf{p}_{\textsf{L}|\varvec{s}}(l). \end{aligned}$$

The prior $\textsf{P}_{\textsf{Z}}$ is assumed to be independent of $\textsf{S}$ and fixed as a standard normal distribution $\textsf{P}_{\textsf{Z}}=\mathcal {N}(\textbf{0},\varvec{I}_d)$. The prior $\textsf{P}_{\textsf{L}|\varvec{s}}$ is set as a log-normal distribution $\textsf{P}_{\textsf{L}|\varvec{s}}=\textrm{LogNormal}(\varvec{l}_{\mu }^{\top }\varvec{s},\varvec{l}_{\sigma ^2}^\top \varvec{s})$. The prior parameters are derived from empirical batch means and variances of the observed log-library sizes. The variational distribution $\textsf{Q}_{\textsf{Z}|\varvec{x},\varvec{s}}$ is chosen as a normal distribution $\mathcal {N}(\varvec{\mu }_{\textsf{Z}},\textrm{diag}(\varvec{\sigma }_{\textsf{Z}}^2))$, and $\textsf{Q}_{\textsf{L}|\varvec{x},\varvec{s}}$ is set as a log-normal distribution $\textrm{LogNormal}(\mu _{\textsf{L}},\sigma _{\textsf{L}}^2)$.

The parameters for these distributions are determined by two encoder neural networks,

3 $$\begin{aligned} f_{\mathsf {enc\, Z}}(\varvec{x,s})=(\varvec{\mu }_{\textsf{Z}},\varvec{\sigma }_{\textsf{Z}})\text { and }f_{\mathsf {enc\, L}}(\varvec{x,s})=(\mu _{\textsf{L}},\sigma _{\textsf{L}}). \end{aligned}$$

scVI obtains latent variables $\varvec{z}$ by sampling from the variational distributions through the reparametrization trick [32].

The sampling distribution $\textsf{P}_{\textsf{X}|\varvec{z},l,\varvec{s}}$ for generating gene-expression data from a given latent variable is assumed to follow a Gamma-Poisson mixture, resulting in a negative binomial distribution. The corresponding decoder network outputs a de-noised gene expression vector that sums to one.

4 $$\begin{aligned} f_{\textsf{dec}}(\varvec{z,s})=\varvec{\rho },\ \sum \limits _{g=1}^N\rho _g=1. \end{aligned}$$

The value $\rho _g$ provides an estimate of the percentage of transcripts in a cell that originate from gene *g*. Gene expression values $x_g$ can be drawn from a negative binomial distribution $\textrm{NB}(l \rho _g, \theta _{g,\varvec{s}})$ parameterized by mean $l \rho _g$ and dispersion $\theta _{g,\varvec{s}}$. The dispersion parameter is constant for every gene across cells of batch $\varvec{s}$. To address dropout, a zero-inflated negative binomial distribution can be used to model count data. The dropout probability parameter $\varvec{\pi }$ is also obtained from the decoder network. The weights of the three neural networks and the parameters $\theta _{g,\varvec{s}}$ are optimized simultaneously by empirically estimating and maximizing the ELBO function

5 $$\begin{aligned} \textrm{ELBO}\left( \varvec{x}, \varvec{s}, \beta \right) & = \textsf{E}_{\textsf{q}_{\textsf{Z,L}|\varvec{x,s}}}\left[ \log \textsf{p}_{\textsf{X}|\varvec{z},l,\varvec{s}}(\varvec{x})\right] \nonumber \\ & \quad -\beta \left( D_{\textsf{KL}}\left[ \left. {\textsf{Q}_{\textsf{Z}|\varvec{x},\varvec{s}}}\right\| {\textsf{P}_{\textsf{Z}}}\right] +D_{\textsf{KL}}\left[ \left. {\textsf{Q}_{\textsf{L}|\varvec{x},\varvec{s}}}\right\| {\textsf{P}_{\textsf{L}|\varvec{s}}}\right] \right) \end{aligned}$$

on mini-batches $\mathbb {M}\subset \mathbb {D}$.

### The scSpecies approach

We consider a scenario involving two scRNA-seq datasets,

6 $$\begin{aligned} \mathbb {D}_{C} = \left\{ \left( \varvec{x}_{C}^{(i)}, \varvec{s}_{C}^{(i)}, c_{C}^{(i)}\right) \right\} _{i=1}^{M_{C}}\ \text {and}\ \mathbb {D}_{T} = \left\{ \left( \varvec{x}_{T}^{(j)}, \varvec{s}_{T}^{(j)}\right) \right\} _{j=1}^{M_{T}}. \end{aligned}$$

Their data points consist of gene expression measurements $\varvec{x}$ and batch indicator variables $\varvec{s}$ from a context species *C* and a target species *T*. Furthermore, context count vectors are clustered into distinct groups based on cell-type labels $c_{C}$, whereas target labels $c_{T}$ are unknown.

The count vectors from both datasets share a gene subset $\varvec{h}$ comprising count values from homologous genes,

7 $$\begin{aligned} \varvec{x}=(\underbrace{x_1,\ldots ,x_H}_{\varvec{h}\text { homologous}},\underbrace{x_{H+1},\ldots ,x_{N}}_{\text {non-homologous}})^{\top }. \end{aligned}$$

The number of non-homologous genes can differ in both datasets, either because a gene has no ortholog in the genome of the other species or because it is not observed within the dataset. Therefore, gene expression vectors can be of different dimension, $N_{C}\ne N_{T}$.

To map both datasets into a unified latent space, we define separate scVI models for each dataset,

8 $$\begin{aligned} \textrm{scVI}^C = \left( f^C_{\textsf{enc Z}}, f^C_{\textsf{enc L}}, f^C_{\textsf{dec}}\right) ,\ \textrm{scVI}^T = \left( f^T_{\textsf{enc Z}}, f^T_{\textsf{enc L}}, f^T_{\textsf{dec}}\right) . \end{aligned}$$

We divide the training procedure for scSpecies into three steps: Training of the context scVI model, followed by an initial data-level nearest-neighbor search and alignment of context and target latent representations.

#### Pre-training on the context dataset

First, the model $\textrm{scVI}^C$ is trained on the context dataset by minimizing its negative ELBO function. Following training, the architecture of the encoder network for the latent variable $\textsf{Z}$ is split up into two parts:

9 $$\begin{aligned} f^C_{\mathsf {enc\, Z}}=f^C_{\textsf{inner}}\circ f^C_{\textsf{outer}}. \end{aligned}$$

The outer part $f^C_{\textsf{outer}}$ consists of the first *L* layer functions and maps data from the input space $\mathcal {X}_C$ to an intermediate feature space $\mathcal {T}$. The inner part, $f^C_{\textsf{inner}}$, consists of the last *M* layers. It encodes an intermediate representation onto the variational parameters with subsequent reparameterization into the latent space $\mathcal {Z}$. We incorporate this inner encoder part into the encoder architecture of $\textrm{scVI}^T$,

10 $$\begin{aligned} f^T_{\mathsf {enc\, Z}}=f^C_{\textsf{inner}}\circ f^T_{\textsf{outer}}. \end{aligned}$$

#### Nearest-neighbor search

When the first layers are initialized randomly, the target model $\textrm{scVI}^T$ cannot leverage the learned structure in its subsequent encoder layers. To leverage the learned weights, we incentivize alignment of intermediate target representations with intermediate features of similar context cells. This leads to an aligned latent space, as layer weights mapping from the intermediate space to the latent space are not updated. To quantify similarity and establish a direct correspondence between cells of the context and target dataset, we perform a nearest-neighbor search on the shared homologous gene subset $\varvec{h}$. The nearest neighbors serve as a set of candidates for every target cell from which the model can choose a best fit to align their intermediate representations during the last training phase.

The nearest-neighbor search identifies an index set $\mathbb {I}_{k}\left( \varvec{x}_{T}^{(j)}\right) \subset \mathbb {I}_{C}$ of *k* nearest neighbors for every target gene count vector $\varvec{x}_{T}^{(j)}$. That is, for every context cell with index $i \in \mathbb {I}_{k}\left( \varvec{x}_{T}^{(j)}\right)$, the chosen measure of association^1^ between the homologous gene counts $\varvec{h}_{C}^{(i)}$ and $\varvec{h}_{T}^{(j)}$ is lower than for cells outside the set:

11 $$\begin{aligned} d_{\textsf{NN}}\left( \varvec{h}_{C}^{(i)},\varvec{h}_{T}^{(j)}\right) \le d_{\textsf{NN}}\left( \varvec{h}_{C}^{(l)},\varvec{h}_{T}^{(j)}\right) \ \text {for all}\ l\in \mathbb {I}_{C}\backslash \mathbb {I}_{k}\left( \varvec{x}_{T}^{(j)}\right) . \end{aligned}$$

Common metrics or distance functions can be used as a measure of association *d* to compare count values of single-cell data. Some popular choices have been investigated in [33]. We utilize cosine similarity, measuring the cosine of the angle between log1p-transformed count vectors, as it is fast to calculate even on datasets containing numerous samples,

12 $$\begin{aligned} d_{\textsf{NN}}\left( \varvec{h}_{C}^{(i)},\varvec{h}_{T}^{(j)}\right) =1-\frac{\left\langle \log \left( \varvec{h}_{C}^{(i)}+1\right) ,\log \left( \varvec{h}_{T}^{(j)}+1\right) \right\rangle }{\left\| \log \left( \varvec{h}_{C}^{(i)}+1\right) \right\| _2\left\| \log \left( \varvec{h}_{T}^{(j)}+1\right) \right\| _2}. \end{aligned}$$

The data-level nearest-neighbor search can also be used to assign preliminary labels. We count the multiplicity of cell labels for all context neighbors and assign the most occurring label as a preliminary prediction,

13 $$\begin{aligned} \hat{c}_{T}^{(j)} = \textrm{mode}\left[ c_{C}^{(i)}: i\in \mathbb {I}_{k}\left( \varvec{x}_{T}^{(j)}\right) \right] . \end{aligned}$$

As the data-level nearest-neighbor search is noisy, we additionally assign agreement scores based on the occurrence of a cell label prediction $\hat{c}_{T}^{(j)}$.

14 $$\begin{aligned} P\left( \hat{c}_{T}^{(j)}\right) = \frac{\left| \left\{ i : c_{C}^{(i)} = \hat{c}_{T}^{(j)} \text { and } i\in \mathbb {I}_{k}\left( \varvec{x}_{T}^{(j)}\right) \right\} \right| }{k} \end{aligned}$$

A higher agreement score indicates lower noise, as there is high agreement among cell labels of the context neighbors. During the following alignment, only target cells exhibiting high agreement scores are considered for alignment in the intermediate space. For this, we collect all agreement scores for target cells predicted to have label $\hat{c}_{T}^{(j)}$ and compute the quantile at level *p* over this set $\left\{ P\left( \hat{c}\right) :\hat{c}=\hat{c}_{T}^{(j)}\right\}$. Finally, we collect the indices of all target cells whose agreement scores of their predicted cell label are higher than the quantile *Q* at level *p*,

15 $$\begin{aligned} \mathbb {J}(p) = \left\{ j: P\left( \hat{c}_{T}^{(j)}\right)> Q\left( p,\left\{ P\left( \hat{c}\right) :\hat{c}=\hat{c}_{T}^{(j)}\right\} \right) \right\} . \end{aligned}$$

#### Aligning the intermediate and latent representations

During alignment, the weights of the pre-trained encoder part $f^C_{\textsf{inner}}$ are not updated. To guide the model towards leveraging the learned structure, scSpecies aligns intermediate representations with high agreement scores

16 $$\begin{aligned} \varvec{t}_{T}^{(j)} = f^T_{\textsf{outer}}\left( \varvec{x}_{T}^{(j)}, \varvec{s}_{T}^{(j)}\right) , j\in \mathbb {J}(p) \end{aligned}$$

with a representation of a suitable context neighbor representation

17 $$\begin{aligned} \varvec{t}_{C}^{(i^*)} = f^C_{\textsf{outer}}\left( \varvec{x}_{C}^{(i^*)}, \varvec{s}_{C}^{(i^*)}\right) , i^*\in \mathbb {I}_{k}\left( \varvec{x}_{T}^{(j)}\right) . \end{aligned}$$

This is facilitated by minimizing the squared Euclidean distance.

18 $$\begin{aligned} \textrm{minimize} \left\| \varvec{t}_{T}^{(j)}-\varvec{t}_{C}^{(i^*)}\right\| _2^2 \text {, if } j\in \mathbb {J}(p). \end{aligned}$$

The optimal choice $i^*\in \mathbb {I}_{k}$ for minimization among the *k* candidates is dynamically determined during the alignment phase: First, we obtain a set of latent context neighbor variables for the target cells considered during alignment,

19 $$\begin{aligned} \mathbb {L}_{k}\left( \varvec{x}_{T}^{(j)}\right) =\left\{ \varvec{z}_{C}^{(i)}: i\in \mathbb {I}_{k}\left( \varvec{x}_{T}^{(j)}\right) \right\} . \end{aligned}$$

These latent variables $\varvec{z}_{C}^{(i)}$ are then decoded with the batch indicator variable $\varvec{s}_{T}^{(j)}$ of their target cell. The target decoder output and target library size $l_{T}^{(j)}$ parameterize a sampling distribution $\textsf{P}_{\textsf{X}|\varvec{z}_{C}^{(i)},l_{T}^{(j)},\varvec{s}_{T}^{(j)}}$, which is used to calculate log density values for every candidate. The cell $i^*$ whose latent representation results in the highest log density value at $\varvec{x}_{T}^{(j)}$ is chosen as the optimal neighbor candidate:

20 $$\begin{aligned} \varvec{z}_{C}^{(i^*)} = \underset{\varvec{z}_{C}^{(i)}\in \mathbb {L}_{k}\left( \varvec{x}_{T}^{(j)}\right) }{\textrm{argmax}} \log \left( \textsf{p}_{\textsf{X}_{T}|\varvec{z}_{C}^{(i)},l_{T}^{(j)},\varvec{s}_{T}^{(j)}}\left( \varvec{x}_{T}^{(j)}\right) \right) . \end{aligned}$$

Using this procedure, it is possible to assign a context neighbor with a fitting cell type if at least one candidate with this cell type is found in this set. The training criterion for the model $\textrm{scVI}^T$ on the target dataset for a data point is

21 $$\begin{aligned} -\textrm{ELBO}\left( \varvec{x}_{T}^{(j)}, \varvec{s}_{T}^{(j)}, \beta \right) +\eta \left\| \varvec{t}_{T}^{(j)}-\varvec{t}_{C}^{(i^*)}\right\| _2^2\left[ j\in \mathbb {J}(p)\right] , \end{aligned}$$

where $[j\in \mathbb {J}(p)]$ is the Iverson bracket that takes value 1 when an index of a target cell *j* is in $\mathbb {J}(p)$, and 0 otherwise. This holds true for cells that exhibited a high degree of agreement during the data-level nearest-neighbor search. As minimization in the intermediate space is only incentivized for cells with these indices, the remaining cells within a mini-batch are grouped around them in a way that minimizes the nELBO of the scVI model.

The scalars $\eta ,\beta \ge 0$ weighing different parts of the loss function, the quantile level $p\in [0,1]$ and the number of nearest neighbors $k\in \mathbb {N}$ are hyperparameters.

#### Measuring the similarity of context and target cells

scSpecies defines a quantitative similarity measure that captures how closely a target cell $\varvec{x}_{T}^{(j)}$ corresponds to a context cell $\varvec{x}_{C}^{(i)}$ of interest. This measure leverages the learned latent manifold, ensuring that it reflects the intrinsic biological features learned by the aligned model. The similarity is computed by first decoding the latent representation of a context cell, $\varvec{z}_{C}^{(i)}$, using the target decoder. This defines a probability distribution for target gene expression profiles. We then evaluate the log-likelihood of the target cell’s observed gene expression vector $\varvec{x}_{T}^{(j)}$ under this distribution. In parallel, the target cell’s own latent representation $\varvec{z}_{T}^{(j)}$ is decoded, and its log-likelihood is calculated. The overall similarity score is defined as the difference of these two log-likelihoods:

22 $$\begin{aligned} d_{\textsf{sim}}\left( \varvec{x}_{C}^{(i)},\varvec{x}_{T}^{(j)}\right) = \log \left( \textsf{p}_{\textsf{X}_{T}\mid \varvec{z}_{C}^{(i)},\,l_{T}^{(j)},\,\varvec{s}_{T}^{(j)}}\left( \varvec{x}_{T}^{(j)}\right) \right) - \log \left( \textsf{p}_{\textsf{X}_{T}\mid \varvec{z}_{T}^{(j)},\,l_{T}^{(j)},\,\varvec{s}_{T}^{(j)}}\left( \varvec{x}_{T}^{(j)}\right) \right) \end{aligned}$$

For a well-aligned latent representation, biologically similar cells will yield similar latent representations, and thus only a small difference in likelihood values. Conversely, for dissimilar context cells, the resulting log-likelihood will be lower, increasing the difference to the target likelihood and therefore reducing the overall similarity score.

We use this similarity measure as a distance metric in a latent nearest-neighbor search, which allows us to transfer cell annotation from the context dataset to target cells. Additionally, this measure provides a basis for assessing the correspondence between context and target cell types by sampling context and target cells of a cell type of interest and calculating the modal value of the obtained similarity value distribution. This indicates which context cell types most closely mirror homologous target cell types.

#### Comparison of gene profiles

To perform a comparison of gene expression profiles between cells of the context and target dataset, we tailor the methods outlined in [19, 34] and [35] to scSpecies. For a latent variable $\varvec{z}$, we obtain normalized gene expression profiles by decoding it with both decoder networks and averaging over all possible batches $\mathbb {S}$:

23 $$\begin{aligned} \varvec{\rho }_{C} = \frac{1}{|\mathbb {S}_{C}|}\sum \limits _{\varvec{s}_{C}\in \mathbb {S}_{C}}f_\textsf{dec}^C(\varvec{z},\varvec{s}_{C}),\ \varvec{\rho }_{T} = \frac{1}{|\mathbb {S}_{T}|}\sum \limits _{\varvec{s}_{T}\in \mathbb {S}_{T}}f_\textsf{dec}^T(\varvec{z},\varvec{s}_{T}) \end{aligned}$$

For a zero-inflated negative binomial model, the normalized expression parameters are multiplied with their respective dropout parameter. Differences in gene expression profiles can be analyzed for homologous genes, for example, by calculating the log2 fold change (LFC) in normalized gene expression parameters

24 $$\begin{aligned} r_{T,C}^g = \log _2\left( \frac{\rho _{T, g}}{\rho _{C, g}}\right) . \end{aligned}$$

For genes *g* with low expression levels but high differences between species, an offset $\varepsilon$ added to the numerator and denominator can maintain a low order of magnitude. When analyzing differences in normalized gene expression, the decoder output layers have to be modified to avoid artifacts from the softmax function. These artifacts can arise due to highly expressed non-homologous genes or due to different data dimensions. We apply the softmax function to homologous and non-homologous genes separately to obtain

25 $$\begin{aligned} \varvec{\rho }_{\textsf{hom}} = \textrm{softmax}(\tilde{\rho }_1,\ldots ,\tilde{\rho }_H),\ \varvec{\rho }_{\textsf{nhom}} = \textrm{softmax}(\tilde{\rho }_{H+1},\ldots ,\tilde{\rho }_N), \end{aligned}$$

where *N* is the dimensionality of the gene expression vector and *H* the number of homologous genes. Afterwards, both vectors are scaled so that they sum to one,

26 $$\begin{aligned} \varvec{\rho } = \left( \frac{H}{N}\varvec{\rho }_{\textsf{hom}}^\top ,\frac{N-H}{N} \varvec{\rho }_{\textsf{nhom}}^\top \right) ^\top . \end{aligned}$$

To analyze gene expression differences within a cell type, we follow the approach of [35], and calculate a mixture distribution of latent states for a cell type $\textsf{C}=c_{T}$.

27 $$\begin{aligned} \textsf{p}_{\textsf{T}}\left( \varvec{z}_{T}\right) =\frac{1}{|\mathbb {C}_{T}(c_{T})|}\sum \limits _{\varvec{x}_{T}^{(i)}\in \mathbb {C}_{T}(c_{T})} \textsf{q}_{\textsf{Z}|\varvec{x_{T}^{(i)},s_{T}^{(i)}}}(\varvec{z}_{T}) \end{aligned}$$

The set $\mathbb {C}_{T}(c_{T})$ is the set of cells with label $c_{T}$ with removed outliers. These outliers are identified by estimating the covariance matrix from variational mean samples $\varvec{\mu }_{T}$. Cells whose variational mean falls outside the 90%-confidence ellipse described by the covariance estimate are removed. When target cell labels are unknown, transferred cell-type labels can be chosen. An LFC distribution of homologous genes for cell types present in both datasets can be estimated by sampling latent variables from $\textsf{P}_{\textsf{T}}$ and computing the corresponding LFC values for normalized and absolute gene expression differences, $r_{T,C}^g$ or $\hat{r}_{T,C}^g$. To calculate the context library size required for absolute gene expression LFC values, it can be imputed by averaging over the nearest context neighbors of the aligned latent representation. We calculate the median of the empirical LFC distribution as well as the probability $P(|r_{T,C}^g|> \delta )$ of observing an LFC in gene *g* higher than level $\delta>0$.

### Layer-wise relevance propagation

In the following, we briefly describe Layer-wise relevance propagation (LRP) [28]. LRP explains the output $f(\varvec{x})$ of a neural network *f* by decomposing it into local contributions of input nodes $x_i$, called relevance scores $R_i(x_i)$ [28]. These relevance scores serve as a measure of each input’s influence on the network’s output: positive scores ($R_i>0$) signify a positive influence, whereas negative scores ($R_i <0$) indicate a negative effect. LRP structurally decomposes the function learned by neural networks into a set of smaller, simpler sub-functions of adjacent layers, while ensuring the conservation of relevance scores across the network. This applies locally, where the sum of the relevance score $R_i$ is conserved across two successive layers of the neural network, and globally between the resulting relevance score for each input node $x_i$ and the output $f(\textbf{x})$ of the model [28].

Considering a neural network with a ReLU activation function, the output $a_k$ of a neuron is given by the input $\hat{a}_j$ of the previous layer and their connected weights $w_{jk}$ of the neurons by

28 $$\begin{aligned} a_k= \max \left( 0,\sum \limits _{j} \hat{a}_j w_{jk}\right) , \end{aligned}$$

including the bias with $\hat{a}_{0}=1$. The relevance scores $R_k$ describe the contribution of each neuron activation $\hat{a}_j$ to $a_k$. They can be computed by the LRP-$\gamma$ rule through

29 $$\begin{aligned} R_j = \sum \limits _{k} \frac{\hat{a}_j (w_{jk} + \gamma w_{jk}^{+}) }{\sum \nolimits _{l} \hat{a}_l (w_{lk} + \gamma w_{lk}^{+})} R_k. \end{aligned}$$

Here, $w_{jk}^{+}$ are the positive weights, while $\gamma$ controls how much these positive contributions are emphasized [36]. LRP methodology aligns with the principles of Deep Taylor Decomposition, which breaks down and redistributes the network’s output function $f(\varvec{x})$ layer by layer through Taylor series expansions. This decomposition allows for the derivation of various LRP rules tailored to the network architecture and the specific function being analyzed [37]. To compute relevance scores for context and target gene expression vectors $\varvec{x}_C, \varvec{x}_T$ we propagate the relevance of their latent variational mean parameters $\varvec{\mu }_C, \varvec{\mu }_T$ through the corresponding encoder network. We aggregate relevance scores through averaging over latent dimensions and data points of a cell type. A direct comparison of scores between species is complicated by the influence of non-homologous genes and batch effects on the relevance scores of homologous genes through the conservation property. Rather, ranked lists of genes by scores can be compared across species.

### Metrics

We evaluated the performance of our method using a range of metrics that assess label transfer, clustering quality, batch and species mixing, and biological conservation. These include the balanced accuracy score (BAS) to assess label transfer in the latent space. Species mixing and biological conservation were evaluated using metrics established by [26, 27]. For species mixing, we replace batch labels with species labels and use the *k*-nearest-neighbor batch effect test (kBET), graph connectivity (GC), and principal component regression (PCR). Biological conservation was assessed with batch average silhouette width (bASW), cell type average silhouette width (cASW), isolation score (Iso), adjusted Rand index (ARI), and normalized mutual information (NMI). More detailed descriptions are provided in Additional file 1: Section *Metrics*.

### Hyperparameters

All scSpecies models were trained with the same neural network architecture. The architecture includes a 10-dimensional latent space, a 300-dimensional intermediate space, and hidden layers with layer normalization, ReLU activation function, and dropout with a rate of $p=0.1$. Gene expression was modeled using a zero-inflated negative binomial distribution with constant dispersion for genes within each experimental batch.

For pre-training and fine-tuning, models were trained for 30 epochs on datasets with more than 10,000 cells and 60 epochs on smaller datasets. The Adam optimizer [38] with standard hyperparameters and a batch size of $M=128$ was used. KL-divergence terms were weighted with $\beta$ incrementally increased from 0.1 to 1 over the first 10 epochs, while the alignment term $\eta$ was raised from 10 to 25 over the first 10 epochs. For nearest-neighbor alignment, the number of neighbors was set to $k=25$, with a quantile cutoff of $p=0.8$ for large datasets and $p=0.5$ for smaller datasets to ensure rare cell types were not underrepresented.

Details on additional training configurations, especially for the comparison models (e.g., scArches, scPoli, and SATURN) are provided in Additional file 1: Section *Hyperparameters*, and Table S4.

### Datasets

We tested our model on publicly available datasets.

The ‘Liver Cell Atlas’ [16, 39] contains a diverse collection of liver cells from multiple species, including mice (both with and without non-alcoholic fatty liver disease), humans, pigs, monkeys, chickens, and hamsters. We utilized all cells acquired through the scRNA-seq and CITE-seq pipelines.

The ‘Single-Cell Atlas of Human and Mouse White Adipose Tissue’ [17, 40] contains gene expression data from human and murine white fat cells. We selected cell samples obtained via single-nucleus sequencing.

The ‘Brain Immune Atlas’ profiles immune response to grade IV glioma. For humans, we selected cells obtained via scRNA-seq of newly diagnosed and recurrent glioblastoma. For mice, we selected cells from the immune response to transplanted glioblastoma [18, 41]. Additional information regarding datasets and their preprocessing can be found in Additional file 1: Section *Preprocessing of Datasets*, Table S5, and Fig. S10.

## Supplementary Information

Additional file 1: Provides explanations of the metrics used, implementation details in Table S4, dataset pre-processing information in Table S5, and additional results in Figs. S1–S10. Table S4: scSpecies network architecture. Table S5: Summary of all datasets used for evaluation. Figure S1: UMAP visualizations of data, intermediate, and latent representations learned by scSpecies and scVI. Figure S2: Reconstructed negative binomial parameters of a standard scVI model and scSpecies. Figure S3: Convergence rates of an unmodified scVI model versus scSpecies during fine-tuning. Figure S4: Heatmaps of the internal similarity measure on the glioblastoma and adipose datasets. Figure S5: UMAP comparison of aligned latent spaces across different alignment methods. Figure S6: UMAP of the latent representation when omitting the nearest-neighbor search during fine-tuning. Figure S7: Scatter plots comparing scSpecies-derived log2 fold change values to data-level analysis values. Figure S8: Differential gene expression analysis comparing mouse and mouse NAFLD samples. Figure S9: Comparison of human and mouse gene LRP scores on the human liver cell atlas. Figure S10: Human liver cell type occurrence among nearest-neighbors across experimental batches.

Additional file 2: Provides the raw values used to generate Fig. 4, organized in a multi-tab Excel file as Tables S1–S3. Table S1: Model performance comparison metrics for various models. Table S2: scSpecies alignment performance for small human liver target dataset. Table S3: scSpecies alignment performance for a reduced homologous gene set.

## Data Availability

The datasets can be accessed via the URLs [39–41]. We provide our workflow as an installable Python package, called *scspecies*. For installation guidelines, tutorial notebooks and more information access the documentation at https://scspecies.readthedocs.io/en/latest/introduction.html and the corresponding GitHub repository [42]. The code used for dataset pre-processing and for generating the results of this publication can be accessed via Zenodo under an MIT license [43].

## Footnotes

1 Lower values indicate higher association.

## References

[1] Leonelli S, Ankeny RA. What makes a model organism? Endeavour. 2013;37(4):209–12. 10.1016/j.endeavour.2013.06.001. 23849606 10.1016/j.endeavour.2013.06.001

[2] Canales CP, Walz K. The mouse, a model organism for biomedical research. In: Walz K, Young JI, editors. Cellular and Animal Models in Human Genomics Research. Cambridge (MA): Academic Press; 2019. pp. 119–40. 10.1016/B978-0-12-816573-7.00006-7.

[3] McMurray F, Moir L, Cox RD. From mice to humans. Curr Diab Rep. 2012;12(6):651–8. 10.1007/s11892-012-0323-2. 22996130 10.1007/s11892-012-0323-2 PMC3488608

[4] Haddad AF, Young JS, Amara D, Berger MS, Raleigh DR, Aghi MK, et al. Mouse models of glioblastoma for the evaluation of novel therapeutic strategies. Neuro-Oncol Adv. 2021;3(1):vdab100. 10.1093/noajnl/vdab100. 10.1093/noajnl/vdab100 PMC8403483 34466804

[5] Lau JKC, Zhang X, Yu J. Animal models of non-alcoholic fatty liver disease: current perspectives and recent advances. J Pathol. 2017;241(1):36–44. 10.1002/path.4829. 27757953 10.1002/path.4829 PMC5215469

[6] Stripecke R, Münz C, Schuringa JJ, Bissig KD, Soper B, Meeham T, et al. Innovations, challenges, and minimal information for standardization of humanized mice. EMBO Mol Med. 2020;12(7):e8662. 10.15252/emmm.201708662. 32578942 10.15252/emmm.201708662 PMC7338801

[7] Cao ZJ, Wei L, Lu S, Yang DC, Gao G. Searching large-scale scRNA-seq databases via unbiased cell embedding with Cell BLAST. Nat Commun. 2020;11(1). 10.1038/s41467-020-17281-7. 10.1038/s41467-020-17281-7 PMC7351785 32651388

[8] Hu J, Li X, Hu G, Lyu Y, Susztak K, Li M. Iterative transfer learning with neural network for clustering and cell type classification in single-cell RNA-seq analysis. Nat Mach Intell. 2020;2(10):607–18. 10.1038/s42256-020-00233-7. 33817554 10.1038/s42256-020-00233-7 PMC8009055

[9] Lotfollahi M, Naghipourfar M, Luecken MD, Khajavi M, Büttner M, Wagenstetter M, et al. Mapping single-cell data to reference atlases by transfer learning. Nat Biotechnol. 2022;40(1):121–30. 10.1038/s41587-021-01001-7. 34462589 10.1038/s41587-021-01001-7 PMC8763644

[10] Donno CD, Hediyeh-Zadeh S, Moinfar AA, Wagenstetter M, Zappia L, Lotfollahi M, et al. Population-level integration of single-cell datasets enables multi-scale analysis across samples. Nat Methods. 2023;20(11):1683–92. 10.1038/s41592-023-02035-2. 37813989 10.1038/s41592-023-02035-2 PMC10630133

[11] Lotfollahi M, Rybakov S, Hrovatin K, Hediyeh-Zadeh S, Talavera-López C, Misharin AV, et al. Biologically informed deep learning to query gene programs in single-cell atlases. Nat Cell Biol. 2023;25(2):337–50. 10.1038/s41556-022-01072-x. 36732632 10.1038/s41556-022-01072-x PMC9928587

[12] Michielsen L, Lotfollahi M, Strobl D, Sikkema L, Reinders MJT, Theis FJ, et al. Single-cell reference mapping to construct and extend cell-type hierarchies. NAR Genom Bioinform. 2023;5(3):lqad070. 10.1093/nargab/lqad070. 37502708 10.1093/nargab/lqad070 PMC10370450

[13] Breschi A, Gingeras TR, Guigó R. Comparative transcriptomics in human and mouse. Nat Rev Genet. 2017;18(7):425–40. 10.1038/nrg.2017.19. 28479595 10.1038/nrg.2017.19 PMC6413734

[14] Rosen Y, Brbić M, Roohani Y, Swanson K, Li Z, Leskovec J. Toward universal cell embeddings: integrating single-cell RNA-seq datasets across species with SATURN. Nat Methods. 2024;21(8):1492–500. 10.1038/s41592-024-02191-z. 38366243 10.1038/s41592-024-02191-z PMC11310084

[15] Sohn K, Lee H, Yan X. Learning Structured Output Representation Using Deep Conditional Generative Models. In: Advances in Neural Information Processing Systems 28. NIPS’15. Montreal: MIT Press; 2015. pp. 3483–91.

[16] Guilliams M, Bonnardel J, Haest B, Vanderborght B, Wagner C, Remmerie A, et al. Spatial proteogenomics reveals distinct and evolutionarily conserved hepatic macrophage niches. Cell. 2022;185(2):379-396.e38. 10.1016/j.cell.2021.12.018. 35021063 10.1016/j.cell.2021.12.018 PMC8809252

[17] Emont MP, Jacobs C, Essene AL, Pant D, Tenen D, Colleluori G, et al. A single-cell atlas of human and mouse white adipose tissue. Nature. 2022;603(7903):926–33. 10.1038/s41586-022-04518-2. 35296864 10.1038/s41586-022-04518-2 PMC9504827

[18] Antunes ARP, Scheyltjens I, Lodi F, Messiaen J, Antoranz A, Duerinck J, et al. Single-cell profiling of myeloid cells in glioblastoma across species and disease stage reveals macrophage competition and specialization. Nat Neurosci. 2021;24(4):595–610. 10.1038/s41593-020-00789-y. 33782623 10.1038/s41593-020-00789-y

[19] Lopez R, Regier J, Cole MB, Jordan MI, Yosef N. Deep generative modeling for single-cell transcriptomics. Nat Methods. 2018;15(12):1053–8. 10.1038/s41592-018-0229-2. 30504886 10.1038/s41592-018-0229-2 PMC6289068

[20] Fernando B, Fromont E, Tuytelaars T. Mining Mid-level Features for Image Classification. Int J Comput Vis. 2014;108(3):186–203. 10.1007/s11263-014-0700-1.

[21] Boureau YL, Bach F, LeCun Y, Ponce J. Learning mid-level features for recognition. In: Proceedings of the 2010 IEEE Computer Society Conference on Computer Vision and Pattern Recognition (CVPR’10). 2010. pp. 2559–66. 10.1109/CVPR.2010.5539963.

[22] Yosinski J, Clune J, Bengio Y, Lipson H. How transferable are features in deep neural networks? In: Advances in Neural Information Processing Systems 27 (NIPS 2014). 2014. pp. 3320–8. 10.48550/arXiv.1411.1792.

[23] McInnes L, Healy J, Melville J. UMAP: Uniform Manifold Approximation and Projection. J Open Source Softw. 2018;3(29):861. 10.21105/joss.00861.

[24] Conde CD, Xu C, Jarvis LB, Rainbow DB, Wells SB, Gomes T, et al. Cross-tissue immune cell analysis reveals tissue-specific features in humans. Science. 2022;376(6594):eabl5197. 10.1126/science.abl5197. 35549406 10.1126/science.abl5197 PMC7612735

[25] Hrovatin K, Moinfar AA, Zappia L, Lapuerta AT, Lengerich B, Kellis M, et al. Integrating single-cell RNA-seq datasets with substantial batch effects. bioRxiv. 2023. 10.1101/2023.03.06.531348. 10.1186/s12864-025-12126-3 PMC12577435 41168710

[26] Luecken MD, Büttner M, Chaichoompu K, Danese A, Interlandi M, Mueller MF, et al. Benchmarking atlas-level data integration in single-cell genomics. Nat Methods. 2022;19(1):41–50. 10.1038/s41592-021-01336-8. 34949812 10.1038/s41592-021-01336-8 PMC8748196

[27] Song Y, Miao Z, Brazma A, Papatheodorou I. Benchmarking strategies for cross-species integration of single-cell RNA sequencing data. Nat Commun. 2023;14(1):6495. 10.1038/s41467-023-41855-w. 37838716 10.1038/s41467-023-41855-w PMC10576752

[28] Bach S, Binder A, Montavon G, Klauschen F, Müller KR, Samek W. On pixel-wise explanations for non-linear classifier decisions by layer-wise relevance propagation. PLoS ONE. 2015;10(7):e0130140. 10.1371/journal.pone.0130140. 26161953 10.1371/journal.pone.0130140 PMC4498753

[29] Keyl P, Bischoff P, Dernbach G, Bockmayr M, Fritz R, Horst D, et al. Single-cell gene regulatory network prediction by explainable AI. Nucleic Acids Res. 2023;51(4):e20. 10.1093/nar/gkac1212. 36629274 10.1093/nar/gkac1212 PMC9976884

[30] Ma XY, Wang JH, Wang JL, Ma CX, Wang XC, Liu FS. Malat1 as an evolutionarily conserved lncRNA plays a positive role in regulating proliferation and maintaining undifferentiated status of early-stage hematopoietic cells. BMC Genomics. 2015;16:676. 10.1186/s12864-015-1881-x. 26335021 10.1186/s12864-015-1881-x PMC4559210

[31] Kim H, Chang W, Chae SJ, Park JE, Seo M, Kim JK. scLENS: data-driven signal detection for unbiased scRNA-seq data analysis. Nat Commun. 2024;15(1):3575. 10.1038/s41467-024-47884-3. 38678050 10.1038/s41467-024-47884-3 PMC11519519

[32] Kingma DP, Welling M. Auto-Encoding Variational Bayes. arXiv preprint. 2013. 10.48550/arXiv.1312.6114.

[33] Skinnider MA, Squair JW, Foster LJ. Evaluating measures of association for single-cell transcriptomics. Nat Methods. 2019;16(5):381–6. 10.1038/s41592-019-0372-4. 30962620 10.1038/s41592-019-0372-4

[34] Boyeau P, Lopez R, Regier J, Gayoso A, Jordan MI, Yosef N. Deep generative models for detecting differential expression in single cells. bioRxiv. 2019. 10.1101/794289. 10.1073/pnas.2209124120 PMC10214125 37192164

[35] Boyeau P, Regier J, Gayoso A, Jordan MI, Lopez R, Yosef N. An empirical Bayes method for differential expression analysis of single cells with deep generative models. Proc Natl Acad Sci U S A. 2023;120(21):e2209124120. 10.1073/pnas.2209124120. 37192164 10.1073/pnas.2209124120 PMC10214125

[36] Montavon G, Binder A, Lapuschkin S, Samek W, Müller KR. Layer-Wise Relevance Propagation: An Overview. In: Explainable AI: Interpreting, Explaining and Visualizing Deep Learning. 2019. pp. 193–209. 10.1007/978-3-030-28954-6_10.

[37] Montavon G, Lapuschkin S, Binder A, Samek W, Müller KR. Explaining nonlinear classification decisions with deep Taylor decomposition. Pattern Recognit. 2017;65:211–22. 10.1016/j.patcog.2016.11.008.

[38] Kingma DP, Ba J. Adam: A method for stochastic optimization. arXiv preprint. 2014. 10.48550/arXiv.1412.6980.

[39] Liver cell atlas. 2022. https://www.livercellatlas.org/. Accessed 20 Jun 2023.

[40] Single-cell atlas of human and mouse white adipose tissue;. https://singlecell.broadinstitute.org/single_cell/study/SCP1376. Accessed 15 Feb 2024.

[41] Brain immune atlas; 2021. https://www.brainimmuneatlas.org/. Accessed 02 Mar 2024.

[42] Schächter C. Code for scSpecies package implementation. GitHub. 2025. Source code available at https://github.com/cschaech/scspecies_package. https://zenodo.org/records/17436208. 10.5281/zenodo.17436207.

[43] Schächter C. Code for scSpecies publication results. Zenodo. 2025. Source code available at https://github.com/cschaech/scspecies_publication. https://zenodo.org/records/17436652. 10.5281/zenodo.17436651.

