# Supplementary material for: scSpecies: enhancement of network architecture alignment in comparative single-cell studies
Source: Genome Biol. 2025 Nov 20;26:397. doi: 10.1186/s13059-025-03866-2 (PMC12636211; doi:10.1186/s13059-025-03866-2)
Supplement: Supplementary file 1 — Additional file 1: Provides explanations of the metrics used, implementation details in Table S4, dataset pre-processing information in Table S5, and additional results in Figs. S1–S10. Table S4: scSpecies network architecture. Table S5: Summary of all datasets used for evaluation. Figure S1: UMAP visualizations of data, intermediate, and latent representations learned by scSpecies and scVI. Figure S2: Reconstructed negative binomial parameters of a standard scVI model and scSpecies. Figure S3: Convergence rates of an unmodified scVI model versus scSpecies during fine-tuning. Figure S4: Heatmaps of the internal similarity measure on the glioblastoma and adipose datasets. Figure S5: UMAP comparison of aligned latent spaces across different alignment methods. Figure S6: UMAP of the latent representation when omitting the nearest-neighbor search during fine-tuning. Figure S7: Scatter plots comparing scSpecies-derived log2 fold change values to data-level analysis values. Figure S8: Differential gene expression analysis comparing mouse and mouse NAFLD samples. Figure S9: Comparison of human and mouse gene LRP scores on the human liver cell atlas. Figure S10: Human liver cell type occurrence among nearest-neighbors across experimental batches. [file 13059_2025_3866_MOESM1_ESM.pdf]

Additional file 1 for scSpecies: enhancement of network architecture alignment in comparative single-cell studies

Clemens Schächter[1, 4, *], Maren Hackenberg[1, 4], Martin Treppner[1], Hanne Raum[2], Joschka Bödecker[2, 3], and Harald Binder[1, 4, 5, *]

[1]Institute of Medical Biometry and Statistics (IMBI), Faculty of Medicine and Medical Center – University of Freiburg, Germany
[2]Neurorobotics Lab, Department of Computer Science – University of Freiburg, Germany
[3]BrainLinks-BrainTools CRIION – Collaborative Research Institute Intelligent Oncology
[4]Freiburg Center for Data Analysis, Modeling and AI – University of Freiburg, Germany
[5]CIBSS, Centre for Integrative Biological Signalling Studies – University of Freiburg, Germany
[*]Corresponding author

October 31, 2025

Supplementary Notes

Metrics

We evaluated species alignment performances with respect to label transfer, cell type clustering quality, and the mixing of species and biology conservation using a combination of established metrics. Below, we summarize each metric and its interpretation.

Label transfer

We measure label transfer accuracy using the Balanced Accuracy Score (BAS). The BAS assesses accuracy while accounting for cell types of different sample sizes within the datasets. For C shared cell types between context and target dataset and confusion matrix entries $\{n_{i,j}\}$ indicating the number of cells from true class i predicted as class j, the BAS is computed as

$$\mathrm{BAS} = \frac{1}{C}\sum_{i=1}^{C}\frac{n_{i,i}}{\sum_{j=1}^{C} n_{i,j}} \in [0,1].$$

Higher BAS indicates more accurate label transfer across all cell types, regardless of their prevalence in the datasets.

Clustering metrics

Adjusted Rand Index (ARI). We utilized the Adjusted Rand index to measure the similarity between two clusterings $\hat{\mathcal{C}}, \mathcal{C}$ (e.g. Leiden clusters and true cell labels).
The Rand Index quantifies the agreement of cell pairs being assigned to the same or different cell types in both clusterings [1],

$$RI = \frac{a+b}{\binom{n}{2}} \in [0,1].$$

In this formula,

$a =$ Number of cell pairs that have the same label in $\mathcal{C}$ and $\hat{\mathcal{C}}$ (true positives),

$b =$ Number of cell pairs that have different labels in $\mathcal{C}$ and $\hat{\mathcal{C}}$ (true negatives),

$n =$ The total number of cells.

The ARI enhances the Rand index by adjusting for chance, considering the expected similarity of all pairings,

$$ARI = \frac{RI - \mathsf{E}[RI]}{\max RI - \mathsf{E}[RI]} \in [-1, 1].$$

An ARI of 1 indicates perfect agreement between true and predicted cell labels, and a value near 0 indicates random label assignments.

Normalized Mutual Information (NMI). Mutual information quantifies the amount of shared information between two clusterings, $\hat{\mathcal{C}}$ and $\mathcal{C}$, and is defined as:

$$MI = \sum_{\hat{c} \in \hat{\mathcal{C}}} \sum_{c \in \mathcal{C}} P(\hat{c}, c) \log \left(\frac{P(\hat{c}, c)}{P(\hat{c})P(c)} \right),$$

where $P(\hat{c})$ and $P(c)$ denote the probabilities of a cell being assigned to cluster $\hat{c}$ in $\hat{\mathcal{C}}$ and to cluster c in $\mathcal{C}$, respectively, and $P(\hat{c}, c)$ is the joint probability of a cell belonging to both clusters. Because mutual information is not bounded, it is normalized using the entropies of the clusterings:

$$NMI = \frac{MI}{\sqrt{H(\hat{\mathcal{C}})H(\mathcal{C})}} \in [0, 1],$$

where the entropy of each clustering is given by

$$H(\hat{\mathcal{C}}) = -\sum_{\hat{c} \in \hat{\mathcal{C}}} P(\hat{c}) \log P(\hat{c}), \quad H(\mathcal{C}) = -\sum_{c \in \mathcal{C}} P(c) \log P(c).$$

Higher values indicate greater similarity between clusterings.

Batch mixing score Batch mixing evaluates how well cells from different experimental batches are intermixed in the learned representation. For each cell, we look at the neighborhood graph with $k = 15$ neighbors and compute the Shannon index to quantify batch diversity. Denoting by p_b the proportion of neighbors belonging to batch b among B total batches, we compute

$$\text{Shannon} \; = \; -\sum_{b=1}^{B} p_b \log(p_b).$$

This value is normalized relative to the expected Shannon index obtained by a random assignment of batch labels and subtracting the result from one, yielding a final score in $[0, 1]$. A value closer to 0 indicates good batch mixing and therefore removal of technical artifacts from a dataset representation.

Alignment quality total score

We measure how well cells from context and target species are aligned within the unified latent space with metrics introduced by [2, 3]. We replace batch label annotation with species labels. The package scib-metrics [2] was used to calculate these metrics. We evaluated species mixing metrics on a random subset of 50,000 cells whenever context and target datasets exceeded this size. Sub-sampling to this size influenced metric scores only by a marginal amount but reduced computational load significantly. Further, to assess metric scores on small context datasets, we sampled the context dataset to contain ten times as many cell as the target dataset cells (10,000 and 100,000 cells), as we observed changing context-target dataset ratios influenced metric scores.

Species mixing metrics

Species mixing metrics measure how well cells from the context and target species are intermixed in the aligned latent representation. All metrics are within the range of 0 and 1, where higher values correspond to better species mixing.

- **Principal Component Regression Comparison** (PCR): Compares the explained variance before and after species integration. For the pre-integrated dataset, we chose 10 principal components of the normalized and log1p-transformed union of the context and target datasets restricted to their shared gene set. Values for using the full gene set were close to zero for almost every method. We excluded this metric for evaluating the performance on a reduced gene set, as the space on which the principal components are calculated changes with the homologous gene set size, which makes values incomparable across datasets.
- **Batch Average Silhouette Width** ($bASW$): Computes a silhouette width comparing each cell to its neighbors of the same species vs. other species.
- **Graph Connectivity** (GC): Measures how well the k-nearest neighbor graph, with $k = 15$, is connected across cell type labels.
- **k-Nearest Neighbor Batch Effect Test** ($kBET$): Tests whether the local distribution of species labels among a cell's $k = 50$ nearest neighbors reflects the global distribution. For evaluating the performance on the small and randomly sampled datasets, we excluded this metric, as we observed its dependence on the ratio between context and target dataset sizes as well as the total cell sample size.

Biology conservation metrics

Biology conservation metrics assess how well biologically meaningful structures, provided by coarse or fine cell type labels, are preserved in the integrated latent space. All metrics are within the range 0 and 1, where higher values correspond to better biology conservation.

- **Normalized Mutual Information** (NMI) / **Adjusted Rand Index** (ARI): The Normalized Mutual Information and Adjusted Rand Index between true cell type labels and Leiden clusters calculated on a $k = 15$ neighborhood graph.
- **Isolation Score** (Iso): Measures how well cell type labels present in only a single species are separated from the rest of the representation using the average silhouette width.
- **Cell-type Average Silhouette Width** ($cASW$): Measures unique cell type isolation using silhouette width with respect to these cell type labels.

Total integrand score

To aggregate the four species mixing and biology conservation scores into a single integrand score (IS), we follow the weighting scheme of [2, 3] and weigh species mixing by 0.4 and biology conservation by 0.6.

$$\begin{aligned} IS &= 0.6BC + 0.4SM \\ &= 0.6\left(\frac{NMI + ARI + Iso + cASW}{4}\right) + 0.4\left(\frac{PCR + bASW + GC + kBET}{4}\right) \end{aligned}$$

Hyperparameters

scSpecies: Models were trained with network architectures listed in Table S4. Gene expression was modeled using a zero-inflated negative binomial distribution with constant dispersion for genes within an experimental batch. We chose a 10-dimensional latent space and a 300-dimensional intermediate space. We pre-trained and fine-tuned models for 30 epochs on datasets with more than 10,000 cells and 60 epochs on datasets with fewer observed samples. Network parameters were updated with the Adam optimizer [4] using standard hyperparameters and a batch size of $M = 128$.

We chose to weigh the KL-Divergence terms with $\beta = 0.1$ at epoch 1, incrementally increasing their influence to $\beta = 1$ over 10 epochs. Similarly, the alignment term started with a weight of $\eta = 10$, which was raised to $\eta = 25$. The number of nearest neighbors was set to $k \in \{1, 25, 250\}$ and the quantile cut-off for alignment was set to $p = 0.8$ across datasets exceeding 10,000 samples. For smaller datasets, we lowered the threshold to $p = 0.5$ to avoid discrimination against scarce cell types. In the latent nearest neighbor search, we pre-computed a set of 200 nearest neighbors for each target cell using the Euclidean distance between variational mean vectors. Among the 25 cells that resulted in the highest likelihood values, we transferred a cell label by majority vote. For differential gene expression analysis, we sampled 10,000 times from the plug-in estimator and set the offset variable to $\varepsilon = 10^{-6}$.
To compute layer-wise relevance scores, we retrained the networks with unbounded ReLU activation functions and without layer normalization, as it is difficult for LRP to handle normalization layers. To counteract exploding intermediate values caused by high gene expression values, we trained the model on log1p-transformed values. We calculated relevance scores using the LRP-γ rule with $\gamma = 0.15$.

For other models, we chose the most similar set of hyperparameters to scSpecies that was achievable with the public implementations of the approaches. These approaches include:

Unintegrated data: As a baseline, no integration or alignment procedure was applied. Normalized and log1p-transformed datasets restricted to the shared gene set were reduced with principal component analysis to 10 dimensions.

Unintegrated scVI: A standard scVI [5] model was trained on the shared gene set of the union of raw context and target datasets.

scArches: scArches [6] with an scVI base model was trained on homologous genes. To accommodate implementation constraints, scArches was trained using a slightly modified architecture consisting of two hidden layers with 256 neurons each.

scPoli: scPoli [7] was initialized with an scVI base model and trained on homologous genes. We used 10-dimensional batch representations, while all other hyperparameters were kept at their default settings.

sysVI: sysVI [8] was also trained on homologous genes. We employed a modified network architecture with two hidden layers with 256 neurons each to best mimic our own framework within the constraints of its public implementation.

SATURN: SATURN [9] was trained with the recommended latent dimension of 256, as a reduction in latent space dimensionality to 10 dimensions decreased model performance. In order to facilitate a comparison, we computed performance metrics on the first 10 principal components extracted from SATURN's 256-dimensional latent space. SATURN was evaluated under two regimes: one using provided target dataset cell labels during metric learning and another where true cell labels were withheld and Leiden clusters (without label matching) were provided instead.

Nearest neighbor search: Label transfer was facilitated on a data level using a nearest neighbor with cosine similarity search on log1p transformed counts. Context cell labels were transferred to a cell by majority vote among the k neighbors.

CellTypist: CellTypist [10] was included as a benchmark for cell type annotation. CellTypist classifiers were trained on normalized and log1p-transformed counts of the context datasets using their default configuration and used for label transfer.

Preprocessing of Datasets

We applied a uniform pre-processing pipeline across all datasets. Initially, the dimension of gene expression vectors of each dataset was reduced to 4000 most highly variable genes [11]. Afterwards, we excluded cells with fewer than 2% nonzero genes or belonging to extremely scarce batch and cell labels with less than 20 samples. We did not exclude scarce batches and cell types when sampling the small liver cell datasets with sample sizes between 1000 and 10000 cells.
Most datasets are annotated with coarse and fine cell type labels. Fine cell type labels are used during training for evaluating metrics and visualization according to the mouse, mouse NAFLD, and human liver cell datasets, as well as the glioblastoma datasets. We use coarse cell type annotation for the white adipose tissue dataset, as the coarse cell type here is equivalent to fine cell type labels on other datasets. We also use coarse cell type labels for constructing the liver cell atlas, as the pig, monkey, chicken, and hamster datasets lack fine cell label annotation. Additional information can be found in Table S5.
In the liver and glioblastoma datasets, some cells have inconsistent cell type labels. For example, some human liver cells are labeled as neutrophils in the fine category and monocytes in the coarse cell label category. We excluded all cells with such a labeling conflict from our analysis.

Supplementary references

1. Vinh NX, Epps J, and Bailey J. Information theoretic measures for clusterings comparison: variants, properties, normalization and correction for chance. J Mach Learn Res 2010; 11:2837–54. DOI: 10.5555/1756006.1953024
2. Luecken MD et al. Benchmarking atlas-level data integration in single-cell genomics. Nat Methods 2022; 19:41–50. DOI: 10.1038/s41592-021-01336-8
3. Song Y, Miao Z, Brazma A, and Papatheodorou I. Benchmarking strategies for cross-species integration of single-cell RNA sequencing data. Nat Commun 2023; 14:6495. DOI: 10.1038/s41467-023-41855-w
4. Kingma DP and Ba J. Adam: A method for stochastic optimization. arXiv preprint 2014. DOI: 10.48550/arXiv.1412.6980
5. Lopez R, Regier J, Cole MB, Jordan MI, and Yosef N. Deep generative modeling for single-cell transcriptomics. Nat Methods 2018; 15:1053–8. DOI: 10.1038/s41592-018-0229-2
6. Lotfollahi M et al. Mapping single-cell data to reference atlases by transfer learning. Nat Biotechnol 2022; 40:121–30. DOI: 10.1038/s41587-021-01001-7
7. Donno CD, Hediyeh-Zadeh S, Moinfar AA, Wagenstetter M, Zappia L, Lotfollahi M, and Theis FJ. Population-level integration of single-cell datasets enables multi-scale analysis across samples. Nat Methods 2023; 20:1683–92. DOI: 10.1038/s41592-023-02035-2
8. Hrovatin K, Moinfar AA, Zappia L, Lapuerta AT, Lengerich B, Kellis M, and Theis FJ. Integrating single-cell RNA-seq datasets with substantial batch effects. bioRxiv 2023. DOI: 10.1101/2023.03.06.531348
9. Rosen Y, Brbić M, Roohani Y, Swanson K, Li Z, and Leskovec J. Toward universal cell embeddings: integrating single-cell RNA-seq datasets across species with SATURN. Nat Methods 2024; 21:1492–500. DOI: 10.1038/s41592-024-02191-z
10. Conde CD et al. Cross-tissue immune cell analysis reveals tissue-specific features in humans. Science 2022; 376:eabl5197. DOI: 10.1126/science.abl5197
11. Satija R, Farrell JA, Gennert D, Schier AF, and Regev A. Spatial reconstruction of single-cell gene expression data. Nat Biotechnol 2015; 33:495–502. DOI: 10.1038/nbt.3192

Supplementary tables

Model	Layer	In	Architecture	Out
f_{outer}	1	$N+S$	$\xrightarrow{\text{Linear, LN, ReLU, Dropout}}$	300
f_{inner}	1	300	$\xrightarrow{\text{Linear, LN, ReLU, Dropout}}$	200
	2	200	$\xrightarrow{\text{Linear}} 2 \cdot 10 \xrightarrow{\text{Rep. trick}}$	10
$f_{\text{enc L}}$	1	$N+S$	$\xrightarrow{\text{Linear, LN, ReLU, Dropout}}$	200
	2	200	$\xrightarrow{\text{Linear}} 2 \cdot 1 \xrightarrow{\text{Rep. trick}}$	1
f_{dec}	1	$10+S$	$\xrightarrow{\text{Linear, LN, ReLU, Dropout}}$	200
	2	200	$\xrightarrow{\text{Linear, LN, ReLU, Dropout}}$	300
	3	300	$\xrightarrow{\text{Linear, (Softmax, Sigmoid)}}$	$2N$
	$\theta_{g,s}$	S	$\xrightarrow{\text{Matrix multiplication}}$	N

Table S4: The network architecture used for the models. N denotes the gene expression data dimension, and S the amount of experimental batch effects in the dataset. Layer functions contain an affine linear transformation, followed by layer normalization (LN), ReLU activation functions that are clipped to the interval $[0, 6]$, and dropout layers with a dropout rate of $p = 0.1$. Latent representations are obtained from the variational mean and scale encoder model output via the reparametrization trick.

Dataset	Organism	Shared genes H	Cells M	Batches S	Number of cell types Coarse	Fine
Liver	C Mouse	4 000	165 680	34	15 (15)	36 (36)
	T Mouse NAFLD	2 860	91 787	22	14 (14)	28 (22)
	T Human	1 808	146 839	30	15 (14)	32 (20)
	T Human small	1 808	5 000	30	15 (14)	32 (20)
	T Pig	1 694	21 907	2	9 (8)	unknown
	T Monkey	1 293	8 483	2	7 (7)	unknown
	T Chicken	1 197	7 456	2	9 (7)	unknown
	T Hamster	1 662	5 955	2	11 (9)	unknown
White adipose tissue	C Mouse	4 000	192 470	26	17 (17)	47 (47)
	T Human	1 937	137 306	24	16 (15)	44 (37)
Glioblastoma	C Mouse	4 000	46 321	6	14 (14)	23 (23)
	T Human	1 823	58 560	12	14 (14)	24 (22)

Table S5: The datasets employed for evaluating scSpecies use mice as context species C. The number H of homologous genes of context and target datasets are listed in the third column. All datasets are annotated with cell type labels, both at coarse and fine levels. The coarse cell type annotation of the adipose tissue dataset corresponds to the fine cell labels of the glioblastoma and liver cell datasets. The amount of distinct labels is detailed in the 'Number of cell types' columns. Additionally, the amount of shared cell types with the context dataset is indicated in parentheses.

Supplementary figures

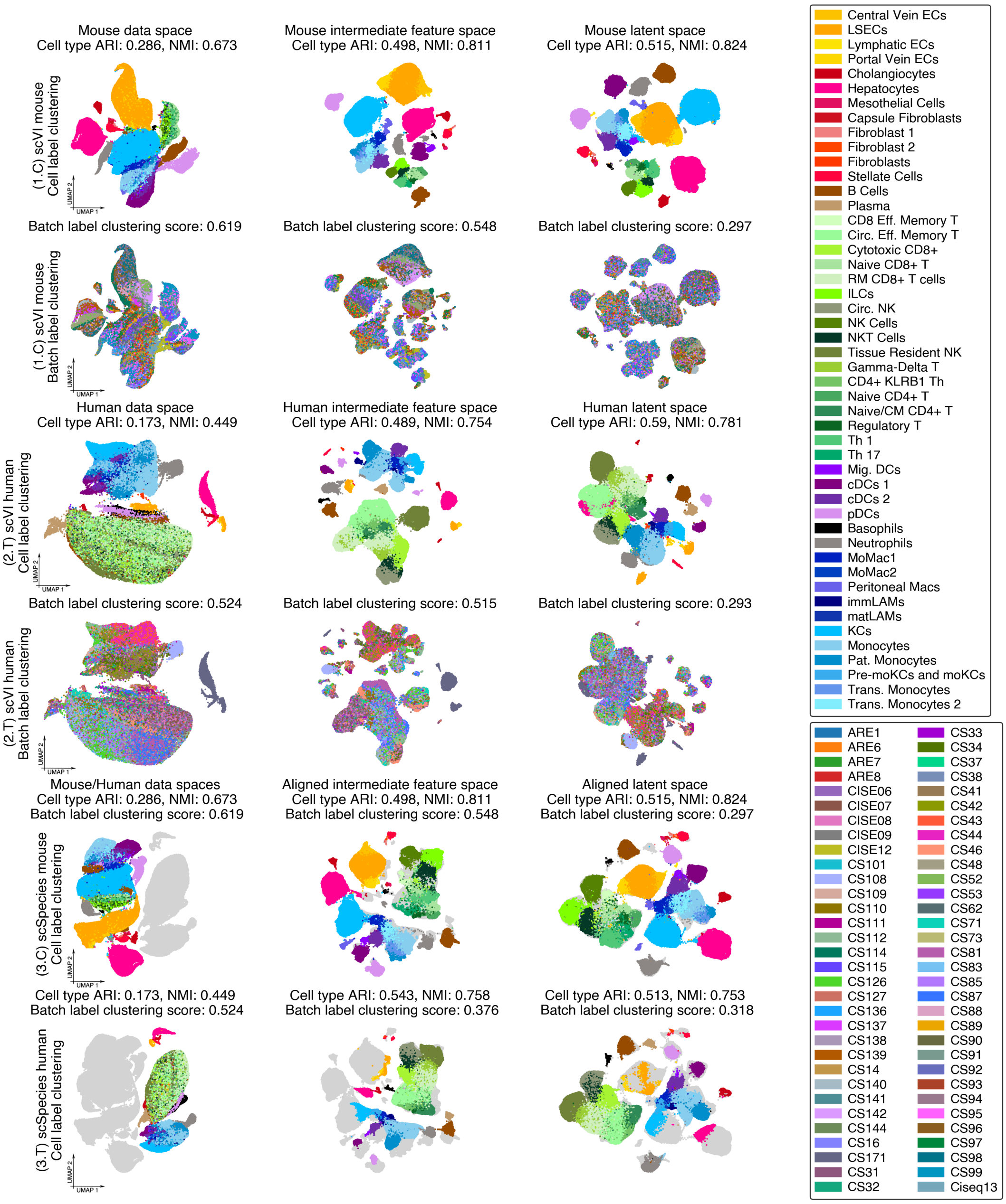

Fig. S1: UMAP visualizations of three data/intermediate and latent representations for the mouse and human liver cell atlas: (1.C) The context (mouse) scVI model, (2.T) a vanilla, unaligned target scVI model trained on human data (middle two rows), and (3.C, 3.T) the aligned dataset representations by scSpecies (bottom two rows). The left, center, and right columns show the 4000-dimensional data space, the 300-dimensional intermediate space, and the 10-dimensional latent space, respectively. Each pair of rows is colored by either cell type or batch labels, as indicated in the row labels. Clustering quality with respect to cell types is measured via ARI and NMI by comparing the true labels with Leiden clusters, whereas batch effects are assessed using a neighborhood-graph-based batch-mixing score. Scores are within the interval $[0, 1]$, and higher values indicate better cluster separation. From the data level to the intermediate space, cell type clustering drastically improves in scVI models, while the technical artifacts of batch effects are removed by mixing cells of different experimental conditions. Aligning mouse and human datasets in the intermediate feature space via scSpecies does not significantly diminish clustering performance for the human dataset, as indicated by similar clustering scores in the aligned and unaligned human latent spaces.

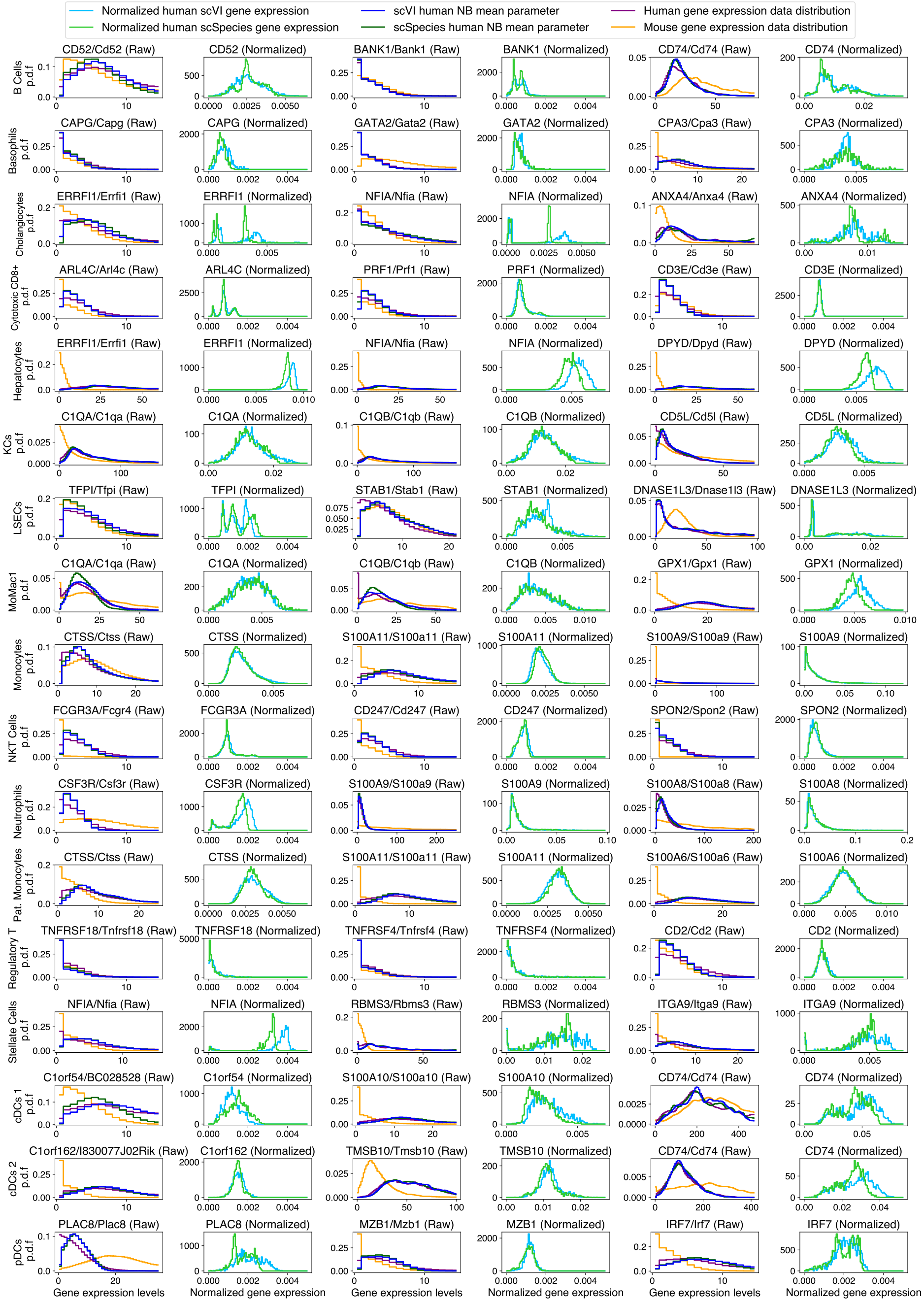

Fig. S2: Comparison of the reconstructed negative binomial mean parameters between a standard scVI model (dark blue) and scSpecies with transferred encoder layers (dark green), alongside the observed data distribution (purple) and corresponding distribution of the homologous mouse gene (orange). The top three marker genes were identified using the Wilcoxon rank-sum test on log-transformed expression values of the common gene set. Observed distributions are displayed for shared fine cell type labels. The reconstructed negative binomial mean parameters track the data distribution of gene expression values, although demonstrating smoothing effect for zero values, as the ZINB decoder distribution mitigates zero inflation. Next to the data-level gene expression distributions are ρ parameter distributions, which normalize gene expression under the modeled true library size for scVI (light blue) and scSpecies (light green). Reconstructed distributions of scSpecies follow those of the standard scVI model, both on the data-level and in the normalized gene expression parameter space, suggesting that the alignment procedure does not negatively impact reconstruction quality.

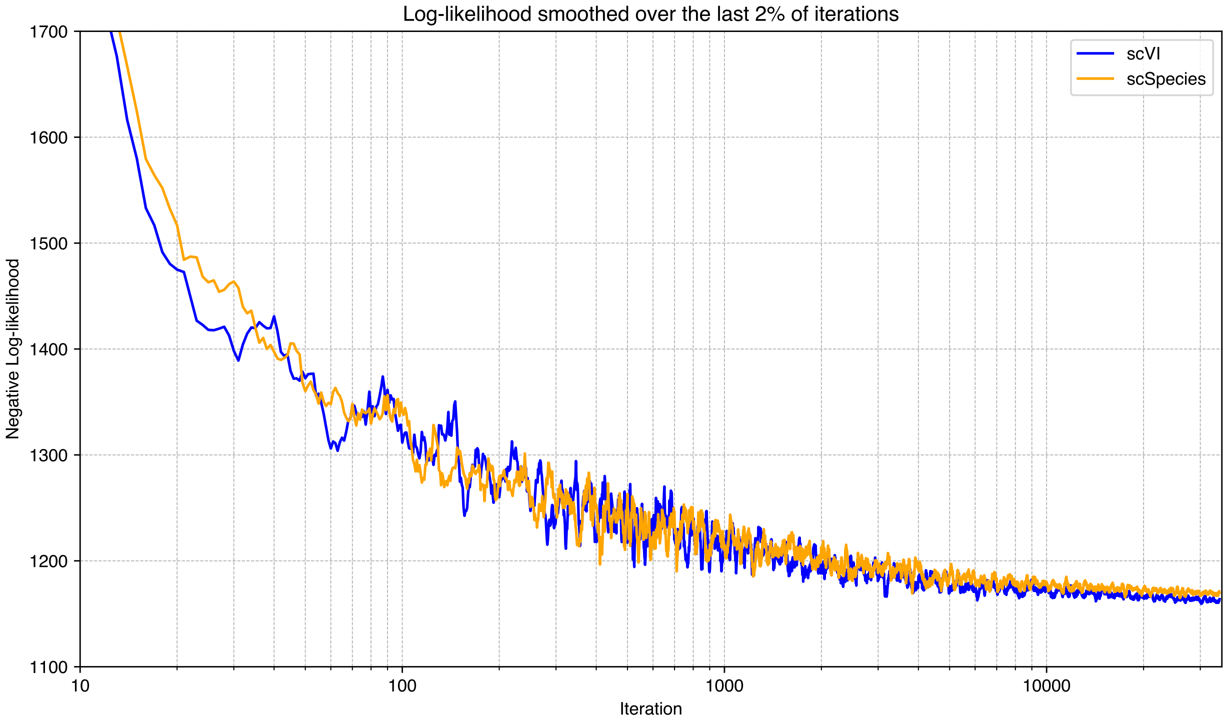

Fig. S3: Reconstruction error of an unmodified scVI model and an scSpecies model with transferred encoder layers during fine-tuning. Results are smoothed over the last 2% of iterations. No major differences in convergence rate or reconstruction quality are observed, indicating that alignment does not negatively impact reconstruction performance.

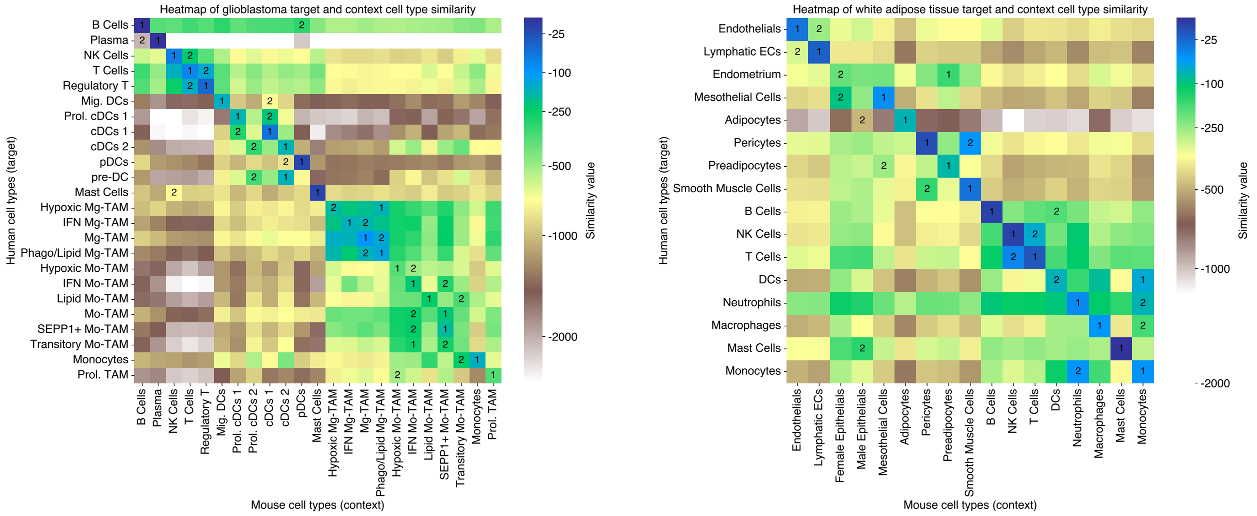

Fig. S4: Heatmap showing similarity values between mouse context and human target cell types in the glioblastoma dataset (left) and the adipose dataset (right). Similarity scores within each target cell type to each context cell type are represented row-wise by colors, and the two most similar context cell types for each target cell type are indicated by numbers.

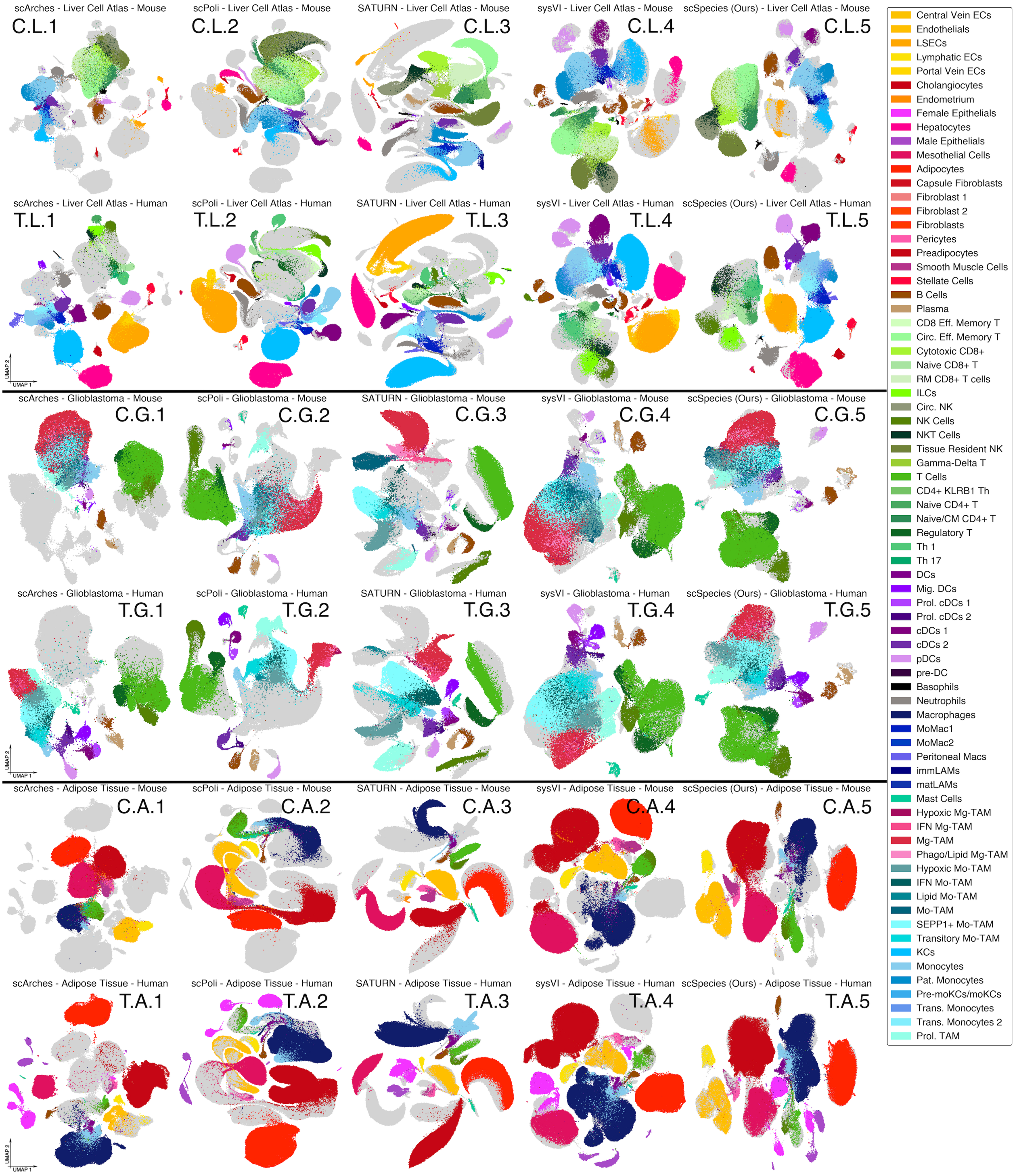

Fig. S5: Comparison of UMAP coordinates for aligned latent spaces across different alignment methods on three datasets: the liver cell atlas (top two rows), the glioblastoma dataset (middle two rows), and the white adipose tissue dataset (bottom two rows). Within each dataset, the mouse context dataset is shown in the top row, while the human target dataset is in the bottom row. Cells from other datasets are displayed in light gray.

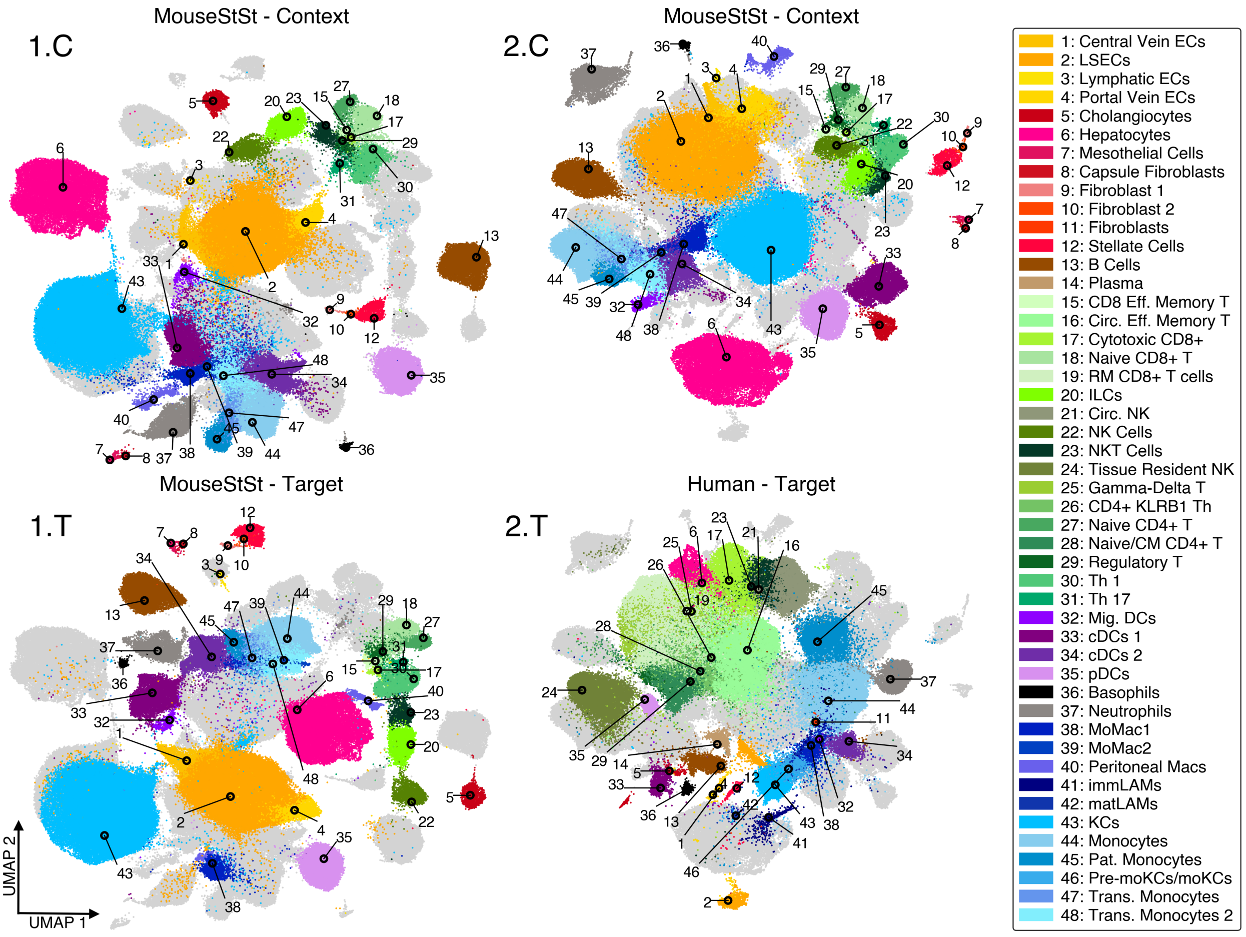

Fig. S6: UMAP coordinates of the latent representation when omitting the nearest neighbor search during fine-tuning. The figure shows the resulting misaligned latent representation of two identical mouse liver cell datasets (1.C, 1.T) and the mouse and human liver cell datasets. (2.C, 2.T) Without guidance by the nearest neighbor search, the target encoder model was unable to leverage the transferred structure and converged to an unaligned latent representation, even when datasets are identical. This results in significantly reduced label transfer accuracy in single-digit accuracy ranges.

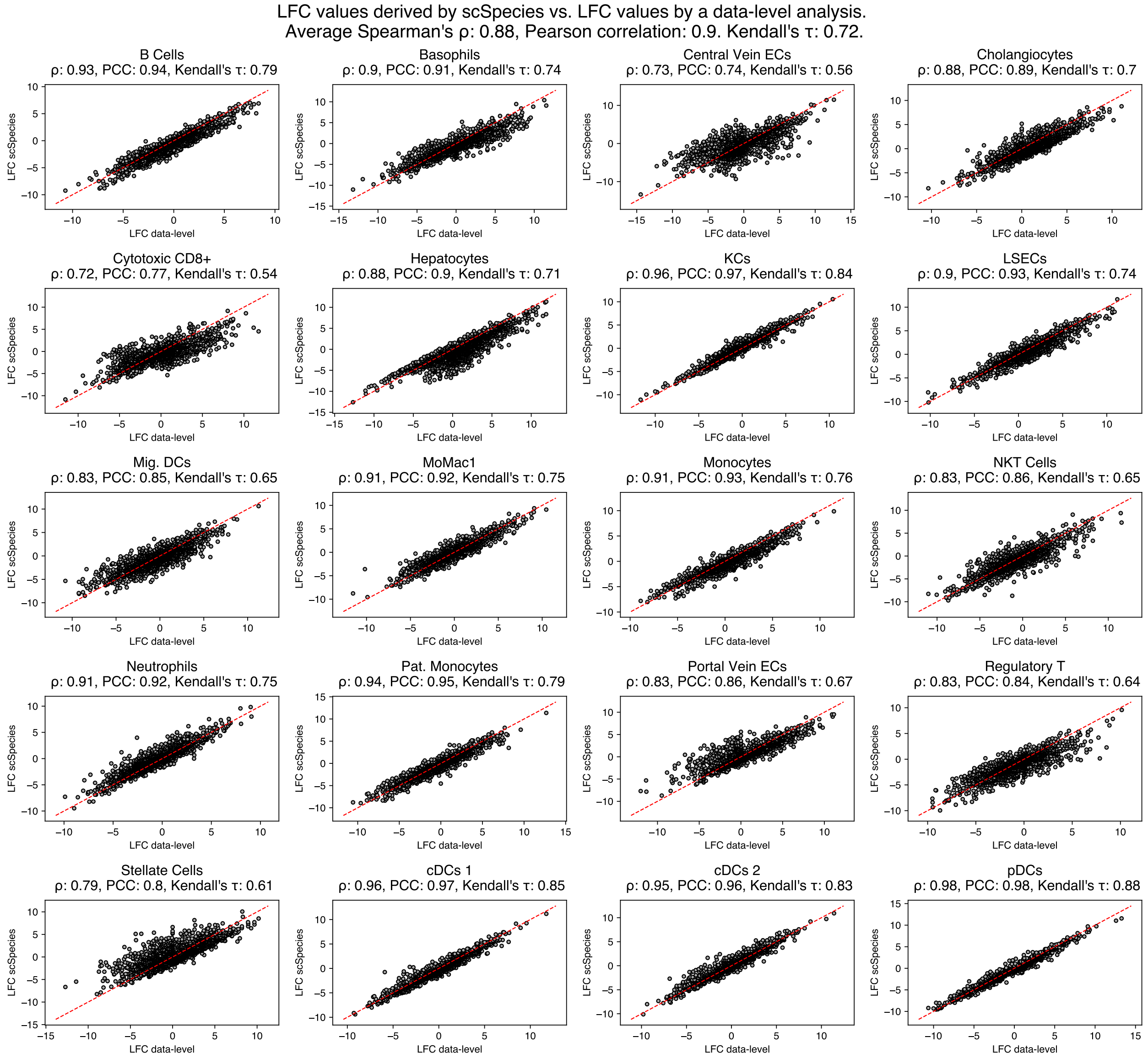

Fig. S7: Comparative analysis of log2 fold change values between human and mouse samples using scSpecies (y-axis) vs. log2 fold change values obtained via data-level analysis on normalized expression profiles (x-axis). Within each shared cell type, we provide Spearman's ρ and Pearson's correlation coefficient between the log2 fold change values of both species and Kendall's τ comparing the ranking of gene lists.

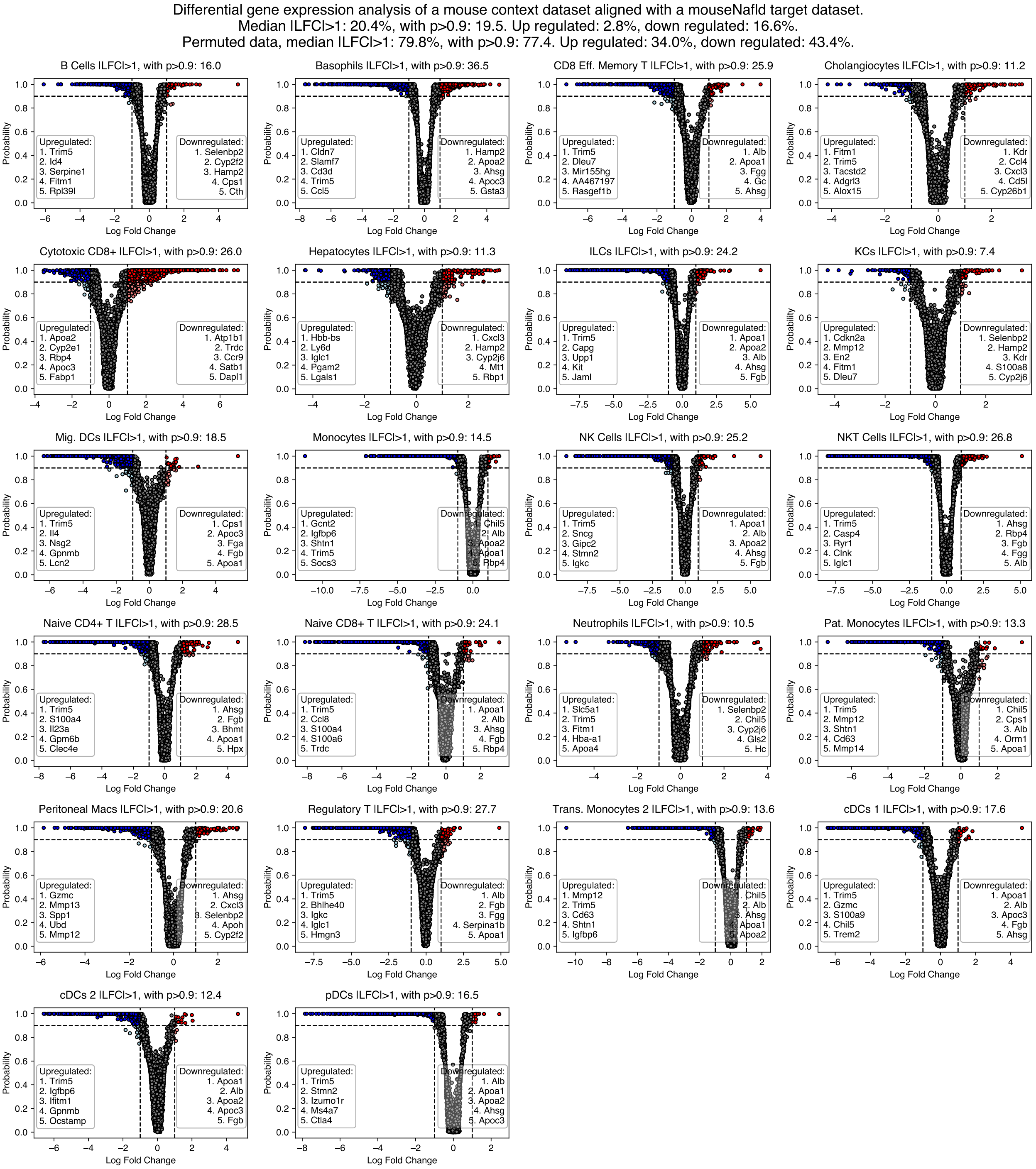

Fig. S8: Comparative analysis of gene expression profiles between mouse and mouse with non-alcoholic fatty liver disease (NAFLD) using scSpecies. The x-axis represents the median of the empirical Log_2fold change distribution, while the y-axis shows the probability of a gene being differentially expressed with an absolute LFC ratio of greater than one within all decoded samples of a cell type. The cell comparisons were derived from a randomly selected latent value within a latent cell type distribution. The figure highlights the top five up-regulated and down-regulated mouse NAFLD genes relative to their healthy mouse counterparts. Compared to our analysis based on the mouse-human genome (as detailed in the results section), we observe a significantly lower percentage of differentially expressed genes.

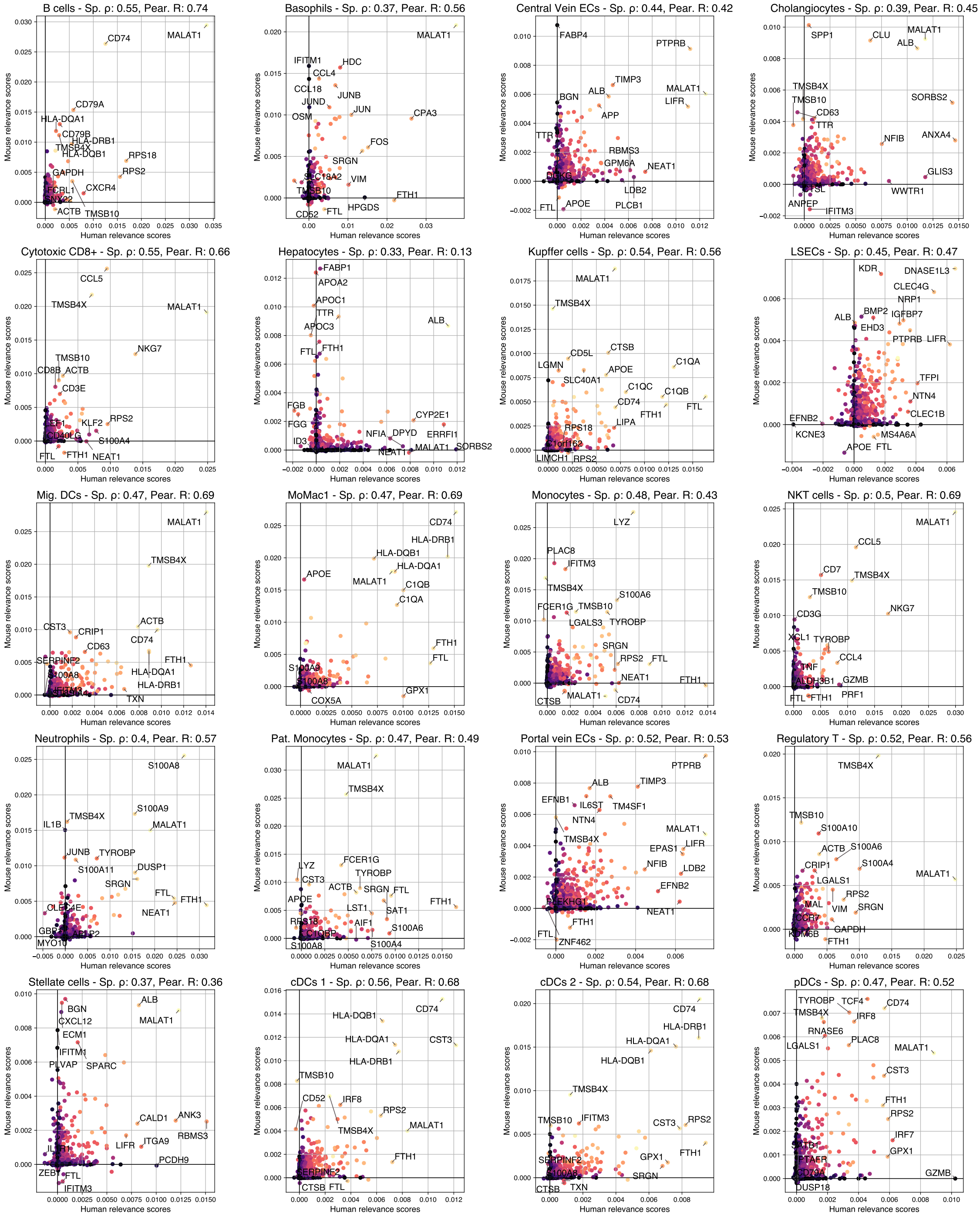

Fig. S9: Plots of human and mouse gene LRP scores compared with each other on the human liver cell atlas. Each dot represents a homologous gene. For every cell, Spearman's ρ and Pearson's correlation coefficient between human and mouse LRP values are given in the axis labels. Coloring corresponds to combined products of human and mouse gene expression, with values of 0 colored in dark tones and high values in bright colors.

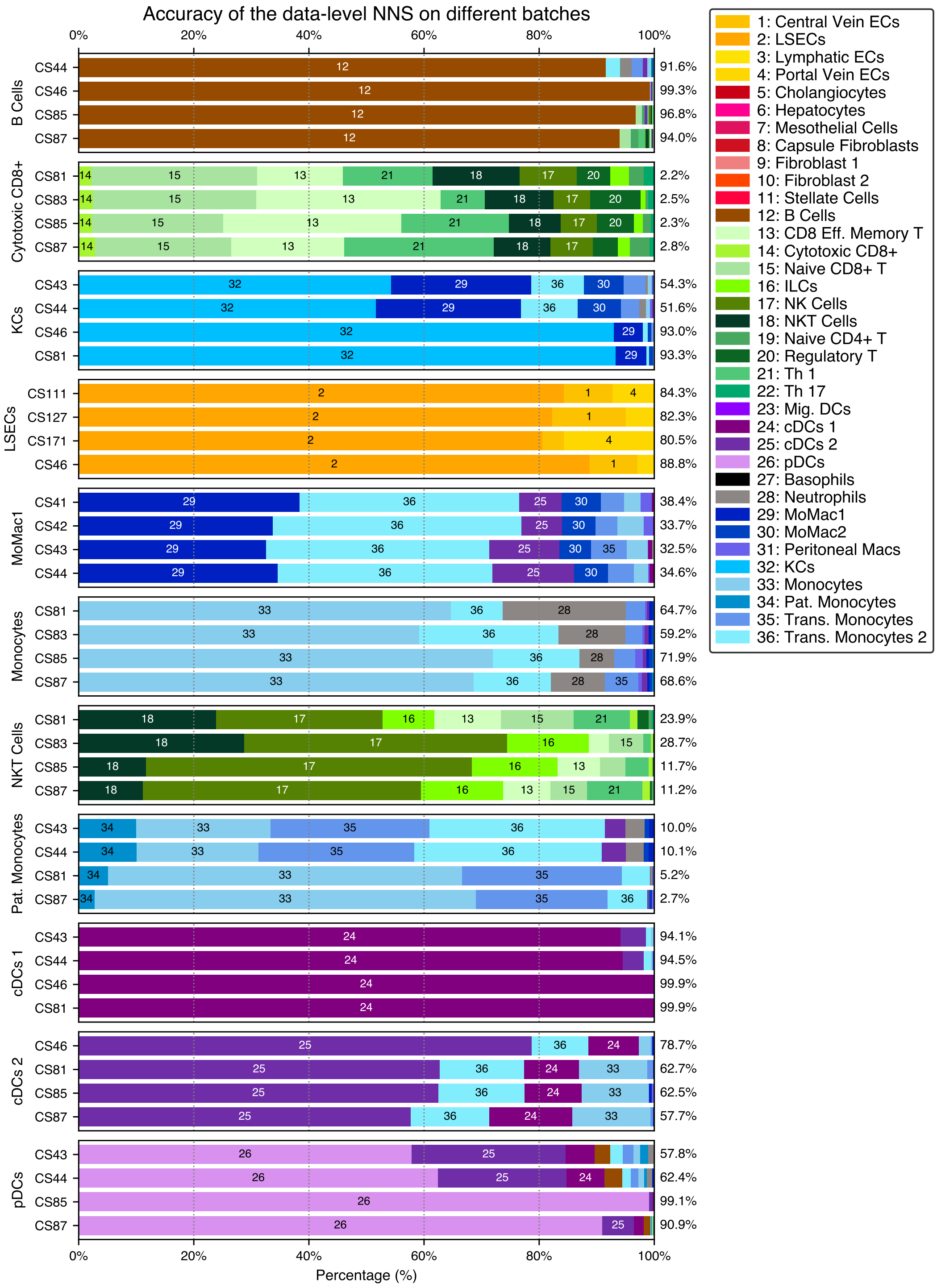

Fig. S10: This figure illustrates how the distribution of context cell types among the $k = 25$ nearest neighbors determined by the data-level NNS varies across experimental batches for human liver cell types. Human cell types with more than four experimental batches with over 200 samples are included in this figure. For each context cell type, four bar plots of the largest experimental batches are shown. Each bar represents an experimental batch and displays the percentage occurrence of context neighbor cell labels. The number codes represent context labels and are explained in the legend. Additionally, the percentage of neighbors matching the true homologous cell type is indicated to the right of each bar. Our findings show that the matching with the true cell type does not vary significantly between experimental batches.
